# Oxyresveratrol Enhances the Anti-Cancer Effect of Cisplatin against Epithelial Ovarian Cancer Cells through Suppressing the Activation of Protein Kinase B (AKT)

**DOI:** 10.3390/biom14091140

**Published:** 2024-09-09

**Authors:** Phatarawat Thaklaewphan, Nitwara Wikan, Saranyapin Potikanond, Wutigri Nimlamool

**Affiliations:** 1 Department of Pharmacology, Faculty of Medicine, Chiang Mai University, Chiang Mai 50200, Thailand; phatarawat.th@gmail.com (P.T.); nitwara.wik@cmu.ac.th (N.W.); saranyapin.p@cmu.ac.th (S.P.); 2 Graduate School, Chiang Mai University, Chiang Mai 50200, Thailand; 3 Lanna Rice Research Center, Chiang Mai University, Chiang Mai 50200, Thailand

**Keywords:** oxyresveratrol, ovarian cancers, PI3K/AKT/mTOR signaling pathway, cisplatin, anti-apoptotic proteins, anti-cancer, natural compounds

## Abstract

Epithelial ovarian carcinoma poses a significant challenge due to its resistance to chemotherapy and propensity for metastasis, thereby reducing the effectiveness of conventional treatments. Hence, the identification of novel compounds capable of augmenting the anti-cancer efficacy of platinum-based chemotherapy is imperative. Oxyresveratrol (OXY), a derivative of resveratrol, has been demonstrated to possess antiproliferative and apoptosis-inducing effects across various cancer cell lines. Notably, OXY appears to exert its effects by inhibiting the PI3K/AKT/mTOR signaling pathway. However, the synergistic potential of OXY in combination with cisplatin against epithelial ovarian cancer has not yet been elucidated. The current study investigated the synergistic effects of OXY and cisplatin on the ovarian cancer cell lines SKOV3 and TOV21G. We found that OXY significantly enhanced cisplatin’s ability to reduce cell viability, induce apoptosis, induce cell cycle arrest, and increase the proportion of cells in the sub-G1 phase. Furthermore, OXY treatment alone dose-dependently inhibited the production of anti-apoptotic proteins including Mcl-1, Bcl-xL, and XIAP under EGF activation. Mechanistically, OXY suppressed the PI3K/AKT/mTOR signaling pathway by reducing phosphorylated AKT, while having no discernible effect on the MAPK pathway. These findings highlight OXY’s potential to enhance ovarian cancer cell sensitivity to chemotherapy, suggesting its development as a pharmaceutical adjunct for clinical use in combination therapies.

## 1. Introduction

Ovarian carcinoma is a gynecological malignancy that is frequently found worldwide, particularly in Northern Europe, Eastern Europe, and Southeast Asia [1]. Although the incidence is falling gradually, ovarian carcinoma is still the cancer with the highest mortality rate among other gynecological malignancies [2]. Epithelial ovarian carcinoma (EOC) is the most common ovarian cancer that responds well to conventional anti-cancer treatments. However, some patients are diagnosed with advanced stages, which carry metastatic characteristics and drug resistance [3,4,5]. Aggressive behaviors in ovarian cancer are associated with genetic mutations that lead to aberrant protein functions. The important protein defects that have been reported include breast cancer gene (BRCA1/2) dysfunction [6], tumor protein 53 (TP53) inactivation [7,8], malfunction of the ataxia telangiectasia mutated (ATM) protein [9,10], or constitutive activity of the mitogen-activated protein kinase (MAPK) pathways [11,12]. Cellular signaling via the phosphatidylinositol 3-kinase (PI3K)/protein kinase B (AKT) signaling pathway, i.e., the fundamental cellular signaling used to regulate cell proliferation, cell growth, and cell survival, has been indicated as the key mechanism of tumorigenesis in various cancers. Typically, PI3K/AKT signal transduction depends on the quantity of endogenous growth factors surrounding the cell’s environment. Nevertheless, in ovarian cancer, abnormalities in signaling mediators, including the overexpression of epithelial growth factor receptor (EGFR) [13], self-activation of EGFR/PI3K/AKT [14,15], and PTEN dysfunction [16], result in the overactivation of signal transduction, excess production of growth and survival-related proteins, and eventually uncontrollable proliferation of anti-cancer drug tolerance in cancer cells [13,17]. The standard approach for managing ovarian cancer involves initial surgery followed by adjuvant therapy, typically consisting of platinum-based chemotherapy alongside anti-tubulin agents such as vinca alkaloids and taxanes [18]. Cisplatin, a commonly used platinum-based chemotherapy, is recommended for ovarian cancer treatment. However, its practical use is limited due to its significant side effects and reduced efficacy against advanced-stage cancer cells. Cancer cells can develop resistance to cisplatin through mechanisms such as increased drug efflux, elevated intracellular components that deactivate cisplatin, enhanced DNA damage repair mechanisms, or the inhibition of apoptosis processes [19]. Certain mutations in cancer cells, such as *p53* or *BRCA2* mutations, can directly impact cisplatin’s effectiveness, leading to increased tolerance to apoptosis [20,21]. Hence, the discovery of new promising agents that have the capability to block the PI3K/AKT pathway, especially when combined with cisplatin, is a key stage in our efforts to reach cancer clearance. Currently, many PI3K/AKT inhibitors are developed and used in clinical practice [22,23]. However, there are limits to their usage, including insufficient efficacy levels and high numbers of side effects. Thus, investigations into searching for natural agents that may be used as alternatives are necessary. 

Oxyresveratrol (2,3′,4,5′-tetrahydroxystilbene, OXY) is a natural stilbene that is mainly found in the genera Morus and Artocarpus, especially in *Morus alba* L. and the heartwood of *Artocarpus lacucha* [24]. OXY contains a wide spectrum of pharmacological activities, including tyrosinase inhibition and antioxidant, anti-inflammatory, neuroprotective, hepatoprotective, antimicrobial, antiviral, and antifungal activities [24]. Particularly for its anti-cancer effect, a few in vitro screening studies have reported that it can decline the cell viability of murine leukemia P-388 cells [25] and several human cancer cell lines, including HepG2, A549, A2780, and MCF-7 [26,27]. Moreover, OXY can induce caspase-dependent cell apoptosis via ROS generation in the triple-negative breast cancer MDA-MB-231 cell line [28], induce cell apoptosis through STAT3 signaling blockage in osteosarcoma Saos-2 cells [29], show a selective cytotoxic effect on the BGC-823 cell line [30], downregulate gene expression and protein levels of vascular endothelial growth factor (VEGF), and inhibit cell growth and migration in a dose-dependent manner in HSC-3, HN-8, and HN-30 cell lines [31]. Previous studies have demonstrated that OXY can inhibit the PI3K/AKT pathway in investigations of anti-inflammatory and anti-psoriasis effects [32,33]. One previous study reports on the inhibitory action of OXY on the PI3K/AKT pathway in HPV-positive cervical cancer cells [34]. However, there is no report pertaining to the regulatory action of OXY on this signal transduction pathway in ovarian cancer cells. Thus, to investigate the anti-cancer effect of OXY as a PI3K/AKT signaling blocker and assess the possibility of its use in clinical practice, we conducted an in vitro study on ovarian cancer cell lines (SKOV3 and TOV21G). Our study suggests that oxyresveratrol is an excellent candidate to be developed as a promising agent targeting PI3K/AKT for use in cancer therapy.

## 2. Materials and Methods

### 2.1. Cell Culture

Human ovarian cancer cell lines (SKOV3 and TOV21G) were purchased from the American Type Culture Collection (Manassas, VA, USA) and kept in liquid nitrogen until the experiment was initiated. RPMI 1640 medium (Gibco, BRL, Norristown, PA, USA) supplemented with 10% fetal bovine serum (Merck KGaA, Darmstadt, Germany) and antibiotics (100 U/mL penicillin and 100 g/mL streptomycin (Thermo Fisher Scientific, Waltham, MA, USA)) was used as complete medium to maintain the cell lines. Cells were cultured in a humidified incubator with a 5% CO_2_ concentration at a temperature of 37 °C. Cell subculturing was performed every 3–5 days when cells were 90% confluent.

### 2.2. Cell Viability Assay

An assessment of the cytotoxicity of OXY was performed by using MTT reagent (3-(4,5-dimethylthiazol-2-yl)-2,5-diphenyltetrazolium bromide), which was purchased from Sigma-Aldrich (Saint Louis, MO, USA). Cells were seeded in a 96-well plate at 1.0–2.0 × 10^4^ cells/well with complete medium overnight to set cell adhesion. After that, cells were exposed to OXY at a series of dilution concentrations, with a maximum concentration of 1000 µM. The agent was subsequently diluted to 0.9766 µM using the 2-fold dilution procedure and left for 48 h. DMSO was used as a vehicle control. After that, old media were aspirated and replaced with MTT 0.4 mg/mL in complete medium for 1 h, and then formazan crystal was dissolved in 100% DMSO. Subjects were measured at 570 nm using a microplate reader (BioTek Instruments, Winooski, VT, USA).

For cytotoxicity assessments of cisplatin and combination, the IC50 of cisplatin was evaluated in the range of 0.1563 to 160 µM. The IC50 was computed using GraphPad Prism 8 (GraphPad Software, Boston, MA, USA). The *IC50* of *OXY* and cisplatin was used to indicate the concentration ratio of OXY and cisplatin used in combination treatment. The combination index was calculated using the following equation:

$$\mathrm{combination}\;\mathrm{index}=\frac{IC50\;cisplatin\;combination}{IC50\;cisplatin\;alone}+\frac{IC50\;OXY\;combination}{IC50\;OXY\;alone}\;.$$

### 2.3. Cell Apoptosis Analysis

Cells were seeded in a 24-well plate overnight in complete medium, then treated with OXY at different concentrations with or without the addition of cisplatin at IC50 concentration, and then left for 48 h. Cells were collected using trypsin (Gibco, BRL, USA), then washed once with PBS before being centrifuged at 3000 rpm for 5 min. Cell pellets were resuspended in annexin-binding buffer and further incubated with annexin V (ImmunoTools, Friesoythe, Germany) and propidium iodide (Sigma-Aldrich, Saint Louis, MO, USA) for 15 min in a dark room. Labeled cells were examined with a DxFLEX flow cytometer (Beckman Coulter, Indianapolis, IN, USA), and the results were analyzed with CytExpert for the DxFLEX program (version 2.0.0.283).

### 2.4. Cell Cycle Analysis

The cell cycle of the treated cells was analyzed using propidium iodide staining and determined with a flow cytometer. Before being treated, the cells were seeded in a 24-well plate with complete medium and left there overnight. For 24 h, OXY was added at various concentrations with or without cisplatin at its IC50. Trypsinization was used to collect the cells for 10 min, and then cells were rinsed once with PBS. Then, cells were fixed and permeabilized with 70% cold ethanol at −20 °C overnight. Before propidium iodide staining, the ethanol was removed by centrifuging the cells at 3000 rpm for 5 min, and then the cells were washed once with PBS and incubated with propidium iodide for 15 min in a dark room. Stained cells were measured using the DxFLEX flow cytometer, and the data were analyzed using CytExpert for the DxFLEX program.

### 2.5. Western Blot Analysis

Cells at a density of 0.2 × 10^6^ per well were seeded in a 24-well plate with complete medium and maintained overnight. The medium was changed into serum-free medium for 24 h before pre-treating the cells with OXY at varying concentrations for 3 h. After pre-treating, cells were activated with EGF at 100 ng/mL for 2–60 min to facilitate determination of the activation status of the early signaling proteins and for 24 h to facilitate anti-apoptosis protein detection. To determine pro-apoptosis proteins, cells were maintained in complete medium and were treated with OXY at different concentrations with or without cisplatin at IC50 for 48 h. After complete treatment, the cells were lysed using reducing sample buffer to obtain cell lysates, which were then stored at −20 °C (after being heated to 95 °C for 5 min). Cell lysates were run through 6–12% gel for protein separation and detection, and then they were transferred onto PVDF membranes (GE Healthcare Life Science, Marlborough, MA, USA). Protein-containing membranes were blocked with 5% bovine serum albumin in TBST and then incubated overnight at 4 °C with primary antibodies. Membranes were incubated with proper secondary antibodies for 2 h at room temperature on an orbital shaker, followed by 5 min TBST washes for three times prior to membrane scanning. Membranes were cleaned three times with TBST, and then the Odyssey^®^ CLx (LI-COR Biosciences, Lincoln, NE, USA) was used to visualize and record immunoreactive bands. Using ImageJ, the band intensity was examined and measured.

### 2.6. Statistical Analysis

The data were recorded as the mean ± standard deviation (SD). A one-way analysis of variance (ANOVA) combined with Tukey’s post hoc multiple-comparisons test was used to analyze the differences between groups. A statistically significant *p*-value was defined as *p* < 0.05 in all analyses, and such values are denoted with asterisks (*) or a hash (#) in this paper. Every experiment was conducted independently at least three times.

## 3. Results

### 3.1. Cytotoxic Effects of Oxyresveratrol (OXY) and Its Combination with Cisplatin on Ovarian Cancer Cells

**Figure 1:**
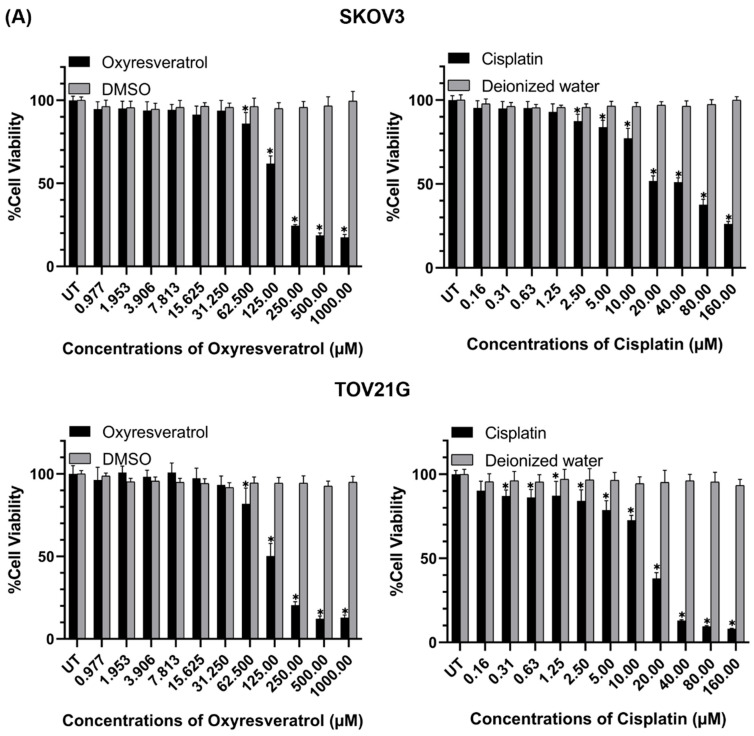

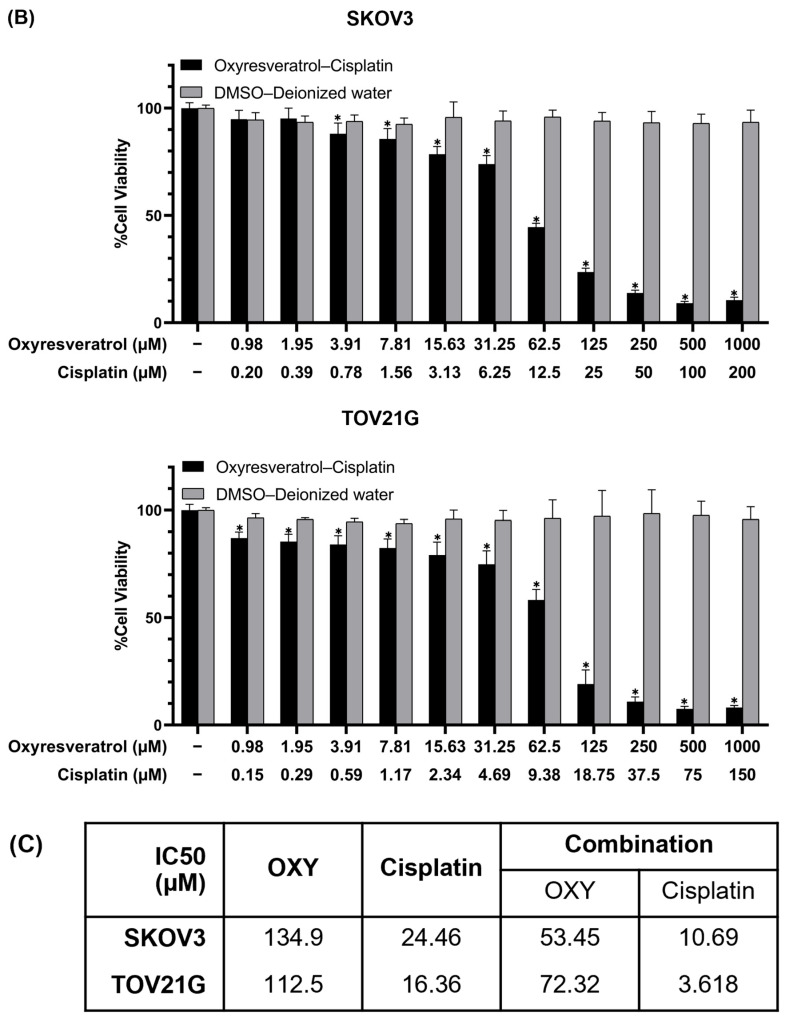
The effects of OXY on the cell viability of SKOV3 and TOV21G ovarian cancer cell lines. (**A**) Percent cell viability of SKOV3 and TOV21G treated with OXY or cisplatin alone for 48 h. (**B**) The combined effect of OXY and cisplatin on ovarian cancer cell viability treated for 48 h. (**C**) The half-maximal inhibitory concentration (IC50) of OXY and cisplatin in individual and combination treatments. Percent cell viability is represented as the mean ± SD of three independent experiments. * *p* < 0.05 compared with the DMSO treatment group.

To determine the cytotoxic effects of oxyresveratrol (OXY) on ovarian cancer cells, SKOV3 and TOV21G cell lines were exposed to OXY in complete media for 48 h, after which cell viability was assessed using the MTT assay. The results demonstrated that OXY exhibited strong cytotoxicity against the ovarian cancer cells, where it diminished the viability of SKOV3 and TOV21G cells in a dose-dependent manner (Figure 1A). The half-maximal inhibitory concentration (IC50) of OXY was 134.90 µM for SKOV3 and 112.50 µM for TOV21G (Figure 1C). To further investigate the cytotoxicity of OXY and cisplatin co-treatment, we first examined the effect of cisplatin on the cell viability of SKOV3 and TOV21G cells and discovered that the viability of SKOV3 and TOV21G cells was gradually decreased when the concentration of cisplatin was increased (Figure 1A). The IC50 values of cisplatin for SKOV3 and TOV21G cells were 24.46 µM and 16.36 µM, respectively (Figure 1C). Treatment of these two cell lines with OXY in combination with cisplatin led to a progressive trend of reductions in cell viability in response to increased concentrations of the combined agents (Figure 1B). In particular, the concentration ratio of OXY to cisplatin in the OXY and cisplatin combination treatment was calculated from their respective IC50 values to be 1:0.20 for SKOV3 and 1:0.15 for TOV21G. Interestingly, the IC50 values of OXY and cisplatin in combination as a treatment for SKOV3 and TOV21G cells were dramatically decreased to 53.45 and 10.69 µM and 72.32 and 3.62 µM, respectively (Figure 1B). The combination index, computed from the IC50 values of individual and combination treatments at a fraction-affected value of 0.5, was 0.833 for SKOV3 and 0.864 for TOV21G cells (Supplementary Figure S1). These findings indicate that OXY and cisplatin treatment synergistically affects cancer cell death.

### 3.2. The Effects of Oxyresveratrol on Ovarian Cancer Cell Apoptosis Induction

**Figure 2:**
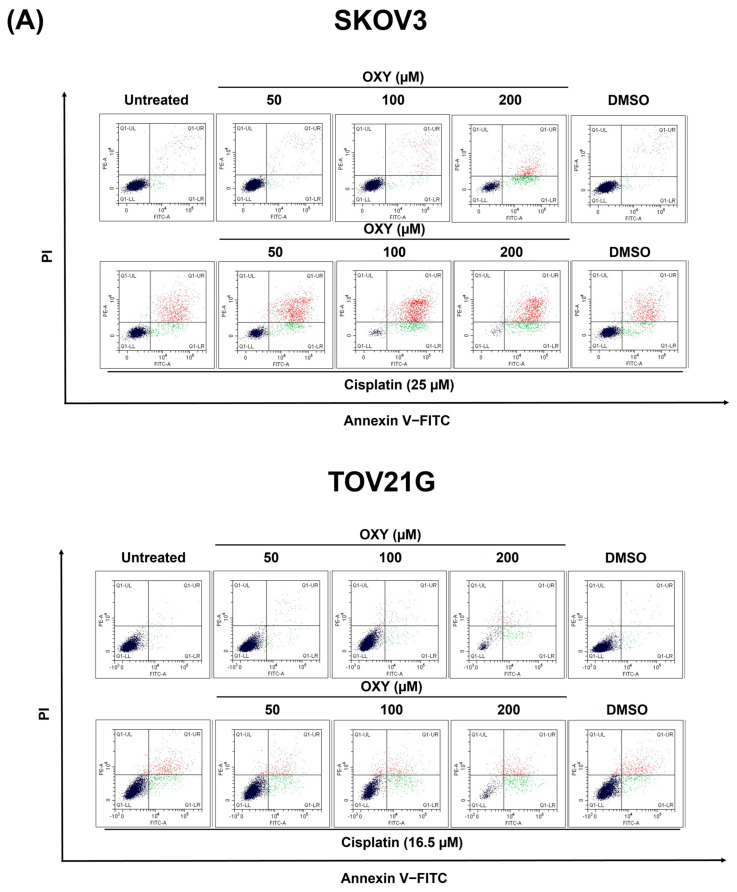

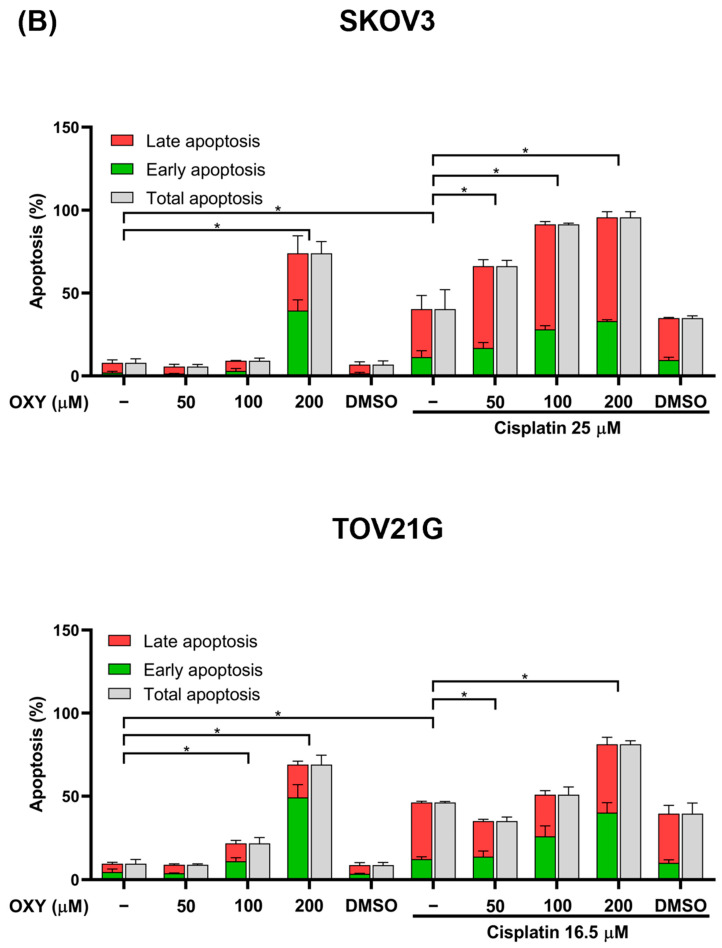

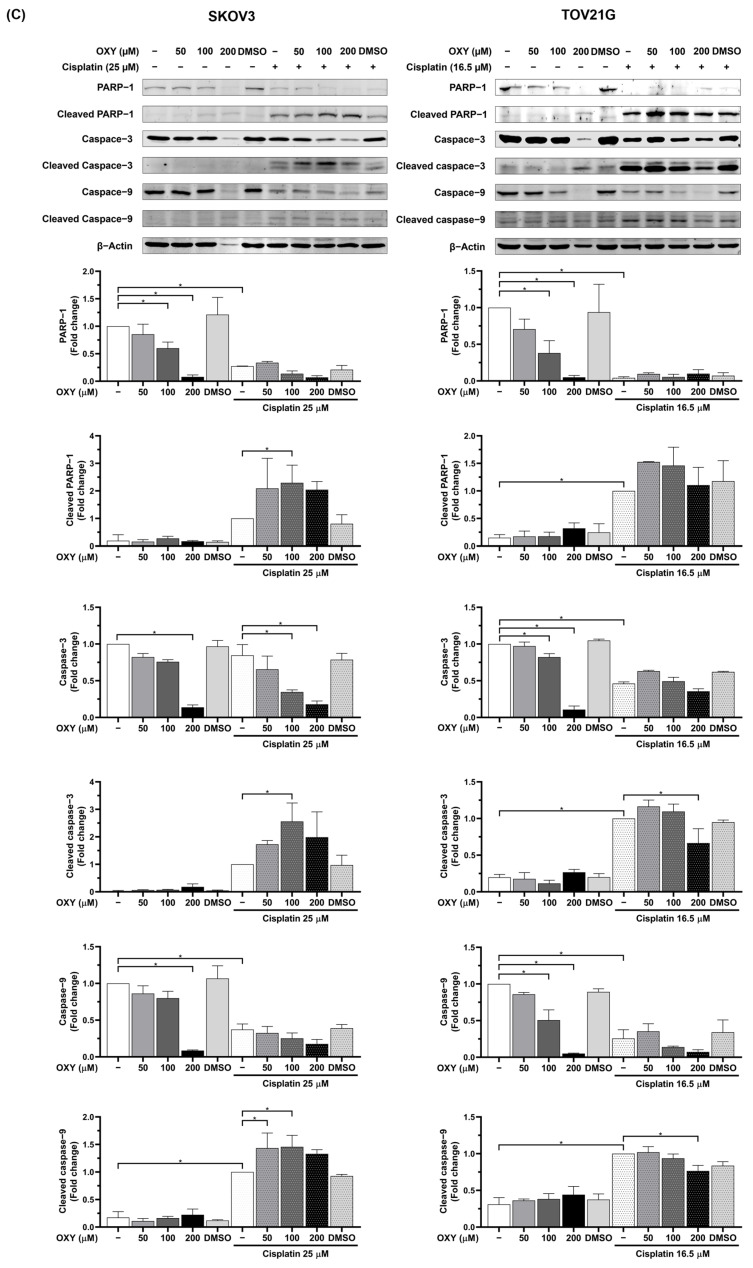
The effects of OXY on ovarian cancer cell apoptosis induction. SKOV3 and TOV21G were exposed to OXY at different concentrations, with or without cisplatin at the IC50 concentration of each cell type, for 48 h. (**A**) Apoptosis cell analysis performed via flow cytometry with annexin V and propidium iodide co-staining where green indicates cells with early apoptosis and red indicates cells with late apoptosis. (**B**) Quantification of early, late, and total apoptosis under different treatment conditions. (**C**) Western blot analysis of PARP-1, caspase-3, caspase-9, and their cleaved forms. Beta actin (β-actin) was used to normalize protein expression. The quantified protein expression levels are represented as the mean ± SD of three independent experiments. * *p* < 0.05. Original images of (**C**) can be found in Supplementary Materials.

To further elucidate the mechanisms underlying the induction of cell death by OXY, we investigated apoptotic cell populations using flow cytometry. SKOV3 and TOV21G cells were treated with varying concentrations of OXY (50, 100, and 200 µM) or cisplatin (25 µM for SKOV3 and 16.5 µM for TOV21G) for 48 h. Subsequently, harvested cells were stained with annexin V and propidium iodide to distinguish early and late apoptotic cells (Figure 2A,B). The results demonstrated that treatment with 200 µM OXY significantly increased apoptosis in both SKOV3 (73.99 ± 5.73%) and TOV21G (68.91 ± 4.60%) cells. Cisplatin treatment induced apoptosis in 40.32 ± 9.57% of SKOV3 cells and 46.24 ± 0.51% of TOV21G cells. The combination of OXY and cisplatin showed a dose-dependent enhancement of apoptosis in SKOV3 cells (66.21 ± 2.88%, 91.46 ± 0.60%, and 95.68 ± 2.72% for OXY at 50, 100, and 200 µM, respectively). In TOV21G cells, the combination treatment of OXY at 200 µM with cisplatin was observed to significantly increase cell death to 81.11 ± 1.70% compared to cisplatin treatment alone.

Western blot analysis was employed to assess the expression levels of protein markers of apoptosis, including PARP-1, caspase-3, and caspase-9, as well as their cleaved forms (Figure 2C). The results revealed that OXY decreased the level of full-length form of PARP-1, caspase-3, and caspase-9 in both cell lines. Consistently, the cleaved forms of these proteins were markedly increased. In SKOV3 cells, cisplatin reduced the levels of full-length PARP-1, caspase 3, and caspase-9 and notably increased their cleaved forms compared to untreated cells. TOV21G cells exhibited a stronger response to cisplatin than SKOV3 cells, showing a substantial decrease in PARP-1, caspase-3, and caspase-9 expression, along with a significant increase in the cleaved forms of these proteins. Notably, an enhanced effect of OXY and cisplatin co-treatment was observed in SKOV3 cells, particularly evident in the reduction in caspase-3 and the augmentation of cleaved PARP-1, cleaved caspase-3, and cleaved caspase-9. However, this enhanced effect was not observed in TOV21G cells.

### 3.3. The Effects of Oxyresveratrol on Ovarian Cancer Cell Cycle Arrest

**Figure 3:**
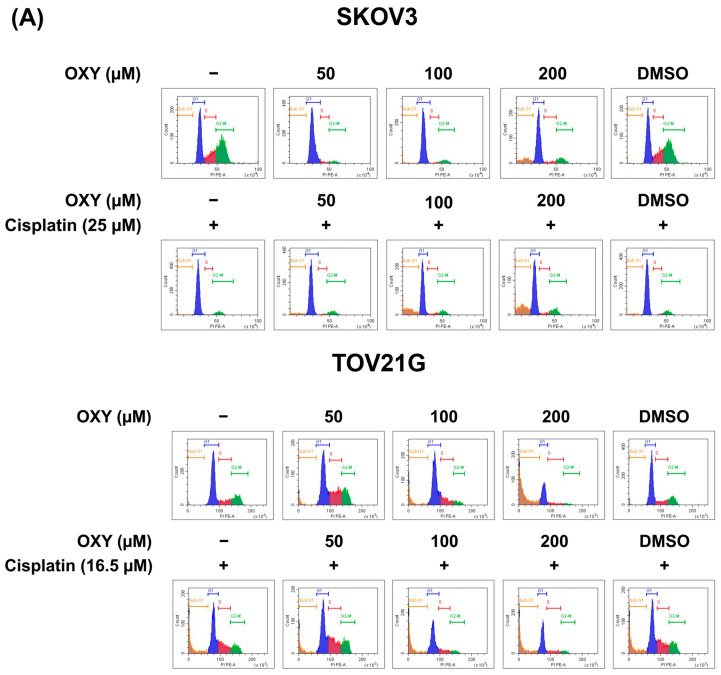

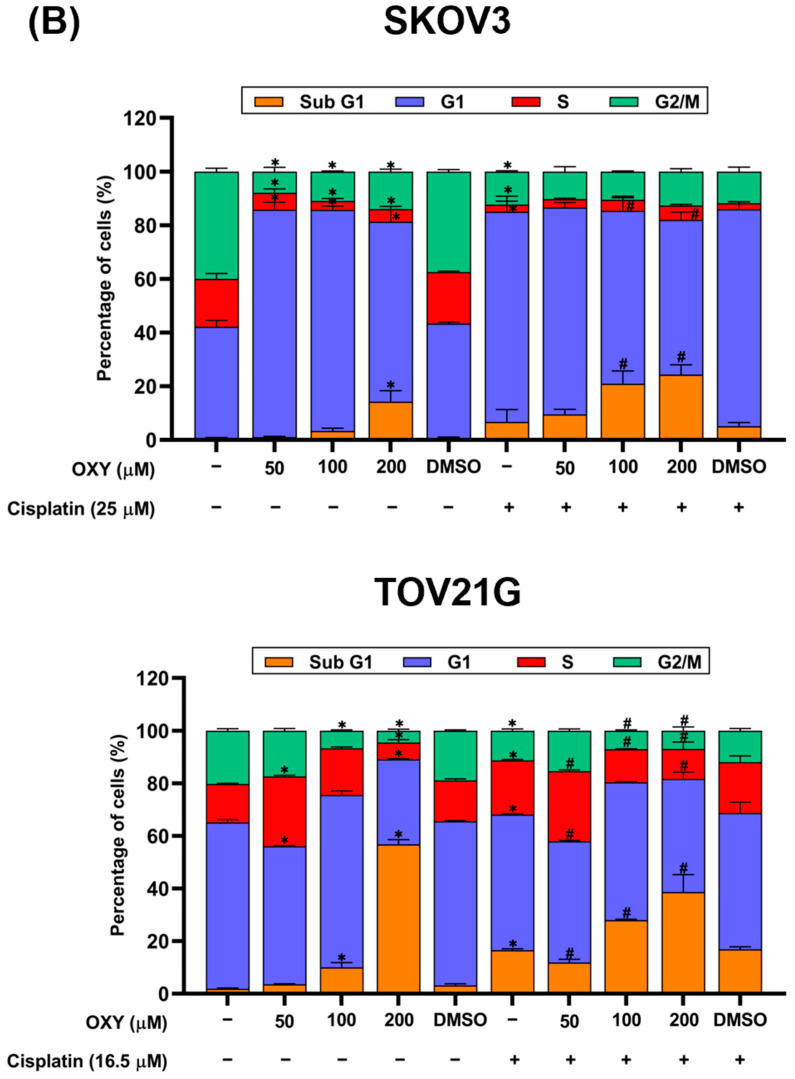
The effects of OXY on cell cycle arrest induction. SKOV3 and TOV21G cells were treated with OXY at various concentrations alone or in combination with cisplatin for 24 h. (**A**) Cell cycle analysis performed by flow cytometry; propidium iodide staining was used to detect the number of DNA strands. (**B**) Qualification of each stage of the cell cycle, with values represented as the mean ± SD of three independent experiments. * *p* < 0.05 vs. the untreated cells. # *p* < 0.05 vs. cisplatin-treated cells.

In previous experiments, OXY demonstrated potential as an anti-cancer agent, despite its precise mechanism of action remaining unclear. Many conventional anti-cancer drugs exert their effects by disrupting the cell cycle, leading to cell cycle arrest and eventual apoptosis induction. Therefore, we investigated the effect of OXY on cell cycle arrest. SKOV3 and TOV21G cells were exposed to OXY at concentrations of 50, 100, and 200 µM, or cisplatin at the IC50 concentration for each cell line, for 24 h. Subsequently, cells were collected and stained with propidium iodide to quantify their DNA content, which was assessed using flow cytometry (Figure 3A,B). The results revealed that OXY induced G1-phase arrest in SKOV3 cells, with a gradual increase in sub-G1 cells with increasing concentration (14.24 ± 4.11% at 200 µM). Similarly, cisplatin arrested cell cycle progression at the G1 phase and increased the number of sub-G1 cells (6.74 ± 4.52%). Interestingly, co-treatment with OXY and cisplatin intensified the effect in a concentration-dependent manner, leading to a gradual increase in sub-G1 cells up to 24.36 ± 3.60% (Figure 3B). In TOV21G cells, S-phase arrest was induced by OXY at 50 µM, and higher concentrations of the agent increased an accumulation of sub-G1 cells. Similarly, cisplatin caused cell cycle arrest in the S phase, with slightly elevated sub-G1 cells. Co-treatment of OXY and cisplatin exhibited the same conditions as OXY treatment alone, alongside a dramatic increase in sub-G1 cells at 100 and 200 µM (27.98 ± 0.37% and 38.66 ± 6.59%, respectively).

### 3.4. The Effects of Oxyresveratrol on Anti-Apoptotic Protein Production

**Figure 4:**
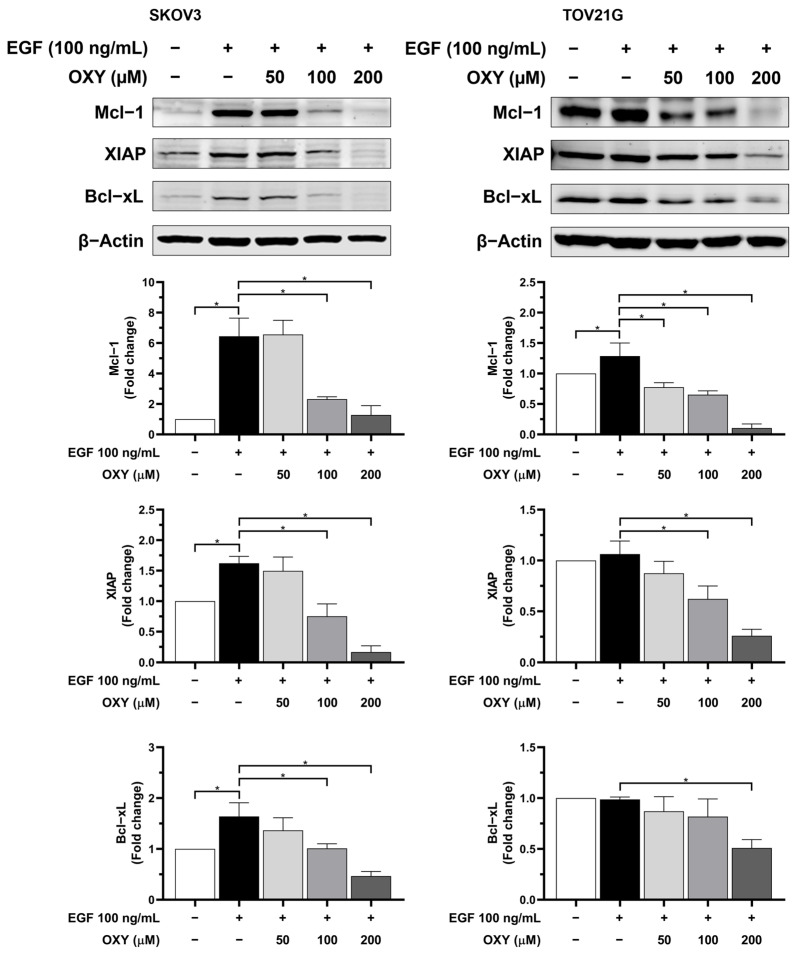
The effects of OXY on the expression level of anti-apoptotic proteins in ovarian cancer cells. SKOV3 and TOV21G cells were pre-treated with OXY for 3 h, then stimulated with EGF at 100 ng/mL for 24 h. The expression of Mcl-1, XIAP, and Bcl-xL was assessed via Western blot analysis and quantified using densitometry. β-actin was used to normalize protein expression. The quantified protein expression levels are represented as the mean ± SD of three independent experiments. * *p* < 0.05. Original images can be found in Supplementary Materials.

Given the potentiation of cisplatin’s effects by OXY in eliminating cancer cells, we hypothesized that OXY might not only induce apoptosis but also reduce the levels of anti-apoptotic proteins, which play a crucial role in preventing apoptosis in many cancer types. Therefore, we further investigated the levels of anti-apoptotic proteins, including Mcl-1, XIAP, and Bcl-xL (Figure 4). SKOV3 and TOV21G cells were pre-treated with OXY at concentrations of 50, 100, and 200 µM for 3 h, followed by stimulation with EGF at 100 ng/mL for 24 h. Cell lysates were collected and analyzed via Western blot analysis. In SCOV3 cells, the levels of Mcl-1, XIAP, and Bcl-xL were significantly upregulated in response to EGF stimulation. Nevertheless, OXY exhibited a concentration-dependent trend in inhibiting the levels of Mcl-1, XIAP, and Bcl-xL. In TOV21G cells, EGF slightly raised the level of Mcl-1, but not XIAP and Bcl-xL, since the basal expression level of these proteins in untreated cells were relatively high. However, OXY could effectively reduce the expression of Mcl-1, XIAP, and Bcl-xL in TOV21G cells even with the presence of EGF (Figure 4).

### 3.5. The Effects of Oxyresveratrol on Inhibiting Cell Growth and Survival Signaling Pathways

**Figure 5:**
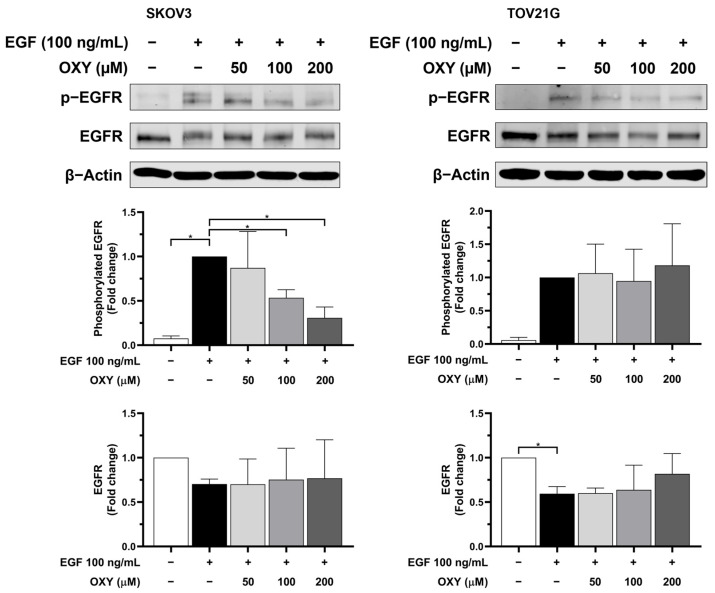
The effects of OXY on the activation of epidermal growth factor receptor (EGFR). SKOV3 and TOV21G cells were pre-treated with OXY at various concentrations for 3 h, followed by 2 min of exposure to 100 ng/mL EGF. The protein expression of phosphorylated EGFR and EGFR was visualized via Western blot analysis, and the protein levels were normalized with β-actin for qualification. The quantified protein expression levels are represented as the mean ± SD of three independent experiments. * *p* < 0.05. Original images can be found in Supplementary Materials.

Typically, cells regulate cell growth and survival through receptors and stimuli, with signals being transmitted via mediator proteins, prompting cells to produce proteins. The AKT and MAPK signaling pathways are well established as crucial for growth and survival, often linked to cancer development. In numerous cancers, mutated receptors and mediator proteins have been found, their dysfunctions being associated with uncontrolled cell proliferation and resistance to apoptosis. In a prior experiment, we noted that OXY significantly reduced the level of anti-apoptotic proteins in ovarian cancer cells, and that it may be possible that OXY could negatively regulate the growth and survival signaling pathways. To identify the molecular targets of OXY in these signaling pathways, we initially determined the phosphorylation level of epidermal growth factor receptor (EGFR) under conditions where OXY was pre-treated for 3 h, followed by EGF stimulation at 100 ng/mL for 2 min. Phosphorylated EGFR occurred robustly in response to EGF stimulation in both SKOV3 and TOV21G cells, accompanied by a decrease in total EGFR during activation (Figure 5). Concentration-dependent responses to OXY were observed only in the SKOV3 cells, where OXY decreased phosphorylated EGFR levels approximately 0.5-fold and 0.3-fold at 100 and 200 µM, respectively, without affecting the total EGFR level.

**Figure 6:**
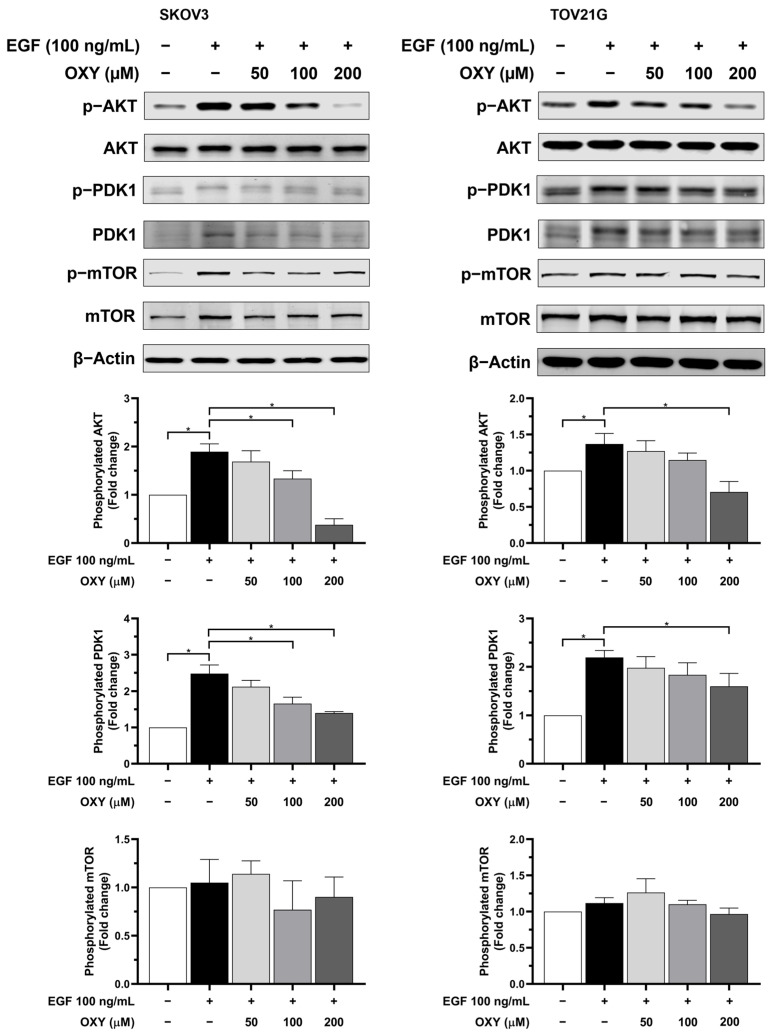
The effects of OXY on PI3K-AKT-mTOR signaling pathway transduction. Cells were pre-treated with OXY at different concentrations for 3 h, then exposed to 100 ng/mL EGF for 30 min. Phosphorylated AKT, PDK1, and mTOR protein levels were assessed via Western blot analysis and normalized with total AKT, β-actin, and total mTOR, respectively. The protein expression levels are represented as the mean ± SD of three independent experiments. * *p* < 0.05. Original images can be found in Supplementary Materials.

Subsequent analyses of important mediators downstream EGFR, including PDK-1, AKT, and mTOR, after 30 min of stimulation (Figure 6) revealed that both SKOV3 and TOV21G cells responded well to EGF. Specifically, in the SKOV3 cells, EGF increased phosphorylated AKT and PDK-1 approximately 1.8- and 2.5-fold, respectively, compared to the untreated cells. In TOV21G, increases were limited to about 1.3- and 2.2-fold increments, respectively. OXY treatment led to a dose-dependent reduction in phosphorylated AKT and PDK-1 in the SKOV3 cells. Specifically, OXY at 100 and 200 µM significantly decreased the amount of phosphorylated AKT around 1.3- and 0.4-fold, and the amount of phosphorylated PDK-1 about 1.7- and 1.4-fold, respectively, compared to the untreated cells. Similarly, in TOV21G cells, OXY at 200 µM also significantly decreased the levels of phosphorylated AKT and PDK-1 roughly 0.7- and 1.6-fold, respectively. However, EGF stimulation and OXY treatment showed no effect on phosphorylated mTOR levels.

**Figure 7:**
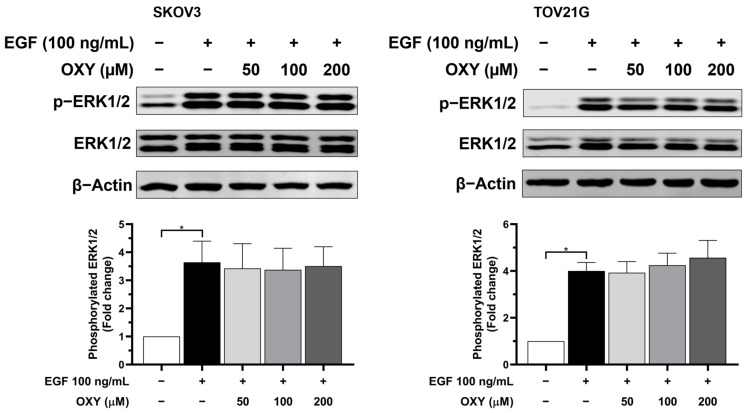
The effects of OXY on MAPK/ERK signaling pathway transduction. Cells were pre-treated with OXY at different concentrations for 3 h, then exposed to 100 ng/mL EGF for 30 min. The phosphorylated ERK1/2 protein level was assessed via Western blot analysis and normalized with total ERK1/2. The protein expression levels are represented as the mean ± SD of three independent experiments. * *p* < 0.05. Original images can be found in Supplementary Materials.

Concurrently, the phosphorylated ERK1/2 level was examined under the same conditions (Figure 7). Phosphorylated ERK1/2 exhibited a significant increase in both SKOV3 and TOV21G after EGF stimulation, with no inhibitory effect being observed from OXY treatment at any concentration.

## 4. Discussion

Currently, the development of medications against cancer represents a remarkable advancement. Numerous medicines have been refined to address the genetic diversity of cancers, aiming for more effective and specific treatments. However, these treatments are inadequate for curing certain cancers, particularly those characterized by rapid growth and high invasiveness. Ovarian cancer, while less common than other types, still exhibits a high mortality rate, with a 5-year survival rate of approximately 30% in developed countries [35]. The primary challenge in treating ovarian cancer is its poor prognosis [36]. In early-stage ovarian cancer, symptoms are often asymptomatic, with few detectable indicators in blood circulation. As a result, most patients are diagnosed at an advanced metastatic stage, complicating treatment due to the cancer’s widespread dissemination and the development of drug resistance, and making it difficult for anti-cancer medications to effectively target and eliminate cancer cells.

The mainstay of conventional treatment for ovarian cancer involves platinum-based chemotherapy, typically combining carboplatin with paclitaxel or docetaxel [18]. These treatments are usually administered following primary surgery to suppress residual cancer cell growth. However, at advanced stages, surgery becomes inappropriate for cancer management, and chemotherapy alone often fails to eradicate cancer cells due to the poor blood supply within dense tumor masses and drug resistance, leading to disease recurrence in some patients. Additional chemotherapeutic approaches such as antimetabolic drugs, hormone therapy, or bevacizumab (for patients with VEGF overexpression) are recommended to enhance cancer removal but offer limited success rates [37,38]. Therefore, the discovery of novel anti-cancer medications that can enhance the efficacy of conventional drugs in advanced stages is crucial for improving treatment outcomes and prolonging patient survival with a good quality of life.

Screening potential compounds from natural sources is a common strategy for discovering new medicines, as many natural compounds are reported to possess anti-cancer effects and show potential as anti-cancer drugs [39,40]. Oxyresveratrol (OXY), a natural stilbene found in various plant species in Southeast Asia [24], is of particular interest. OXY is a derivative of resveratrol, which has been extensively studied for its anti-cancer effects [40]. Previous studies have shown that OXY exerts greater antiproliferative effects on bladder cancer T24 cells compared to resveratrol and other derivatives [41]. In studies involving highly chemo-resistant, triple-negative human breast cancer cell lines like MDA-MB-231, OXY induced apoptosis-like characteristics by generating reactive oxygen species, resulting in mitochondrial membrane depolarization, the release of apoptosis-inducing factors, and, ultimately, DNA fragmentation [28]. Furthermore, OXY modulated gene expressions associated with apoptosis, cell cycle modulation, and DNA repair in breast cancer MCF-7 cells [42]. In studies involving human head and neck squamous cell cancer under deferoxamine-induced hypoxia, OXY pre-treatment inhibited cancer stem cell markers such as Oct-4, Nanog, CD-44, and CD-105, suggesting that OXY may prevent the epithelial–mesenchymal transition (EMT) and metastasis [43]. Additionally, a study in human colon cancer HT-29 cells found that OXY reduced Snail/E-cadherin expression during TGF-β activation and interfered with EMT-related miRNA expression [44]. Further studies on the molecular mechanism of OXY have revealed its attenuation of STAT3 signaling in Saos-2 and U2OS cells [29,45].

In the current study, we aimed to determine the anti-cancer effects of OXY, identify its exact molecular mechanism, and explore the combined effects of OXY and conventional chemotherapy (cisplatin) on ovarian cancer SKOV3 and TOV21G cells. Cell viability analysis results demonstrated that treatment with OXY or cisplatin alone reduced cell viability by a certain percentage. As expected, co-treatment with OXY and cisplatin significantly enhanced the reduction in cell viability, with the combination’s IC50 for both cell lines decreasing by more than half in comparison to individual exposures. The combination index at each tested fraction was less than one, indicating that OXY synergistically intensifies the effect of cisplatin. In short, OXY not only induces cell death directly but also sensitizes cancer cells to chemotherapy. The enhancement of OXY’s anti-cancer effects on conventional chemotherapy was consistent with previous studies, which showed that OXY combined with doxorubicin promoted dose-dependent reductions in cell viability in breast and lung cancer cells [46,47]. Furthermore, the combination of OXY and dacarbazine synergistically induced cell cycle arrest and apoptosis in WM-266-4 cells [48].

Flow cytometry analysis with annexin V and propidium iodide co-staining clearly demonstrated that OXY significantly reduced total cell numbers and increased the number of apoptotic cells, particularly at high doses, in both SKOV3 and TOV21G cells. At the IC50 of cisplatin, there was a moderate increase in apoptosis levels, while the addition of OXY enhanced cisplatin’s effects in a dose-dependent manner in SKOV3 cells. However, this trend was less pronounced in TOV21G cells, where the enhancement was observed only at higher doses. Furthermore, Western blot analysis showed that OXY and cisplatin induced cell apoptosis via caspase-dependent pathways by reducing full-length caspase-3, caspase-9, and PARP-1 and increasing their cleaved forms. However, differences were observed in how the SKOV3 and TOV21G cells responded to OXY. In particular, there was an increase in the cleaved forms of PARP-1, caspase-3, and caspase-9 following OXY treatment in cisplatin-treated SKOV3 cells but not in cisplatin-treated TOV21G cells. These results suggest that OXY was able to sensitize SKOV3 cells to cisplatin more effectively than TOV21G cells. Compared to SKOV3 cells, it is possible that TOV21G cells may contain certain active molecular players in the survival signaling pathway that make these cancer cells less sensitive to OXY–cisplatin treatment.

To further investigate the effects of OXY on cell proliferation and survival, we examined cell cycle interference by measuring the DNA content via flow cytometry. In SKOV3 cells, OXY alone or cisplatin alone induced cell cycle arrest, particularly at the G1 phase. OXY–cisplatin co-treatment increased the sub-G1 population in a concentration-dependent manner, indicating that the cells were sensitized to death. In contrast, OXY alone or cisplatin alone induced S-phase arrest in the TOV21G cells. Similar to the observation in SKOV3 cells, combinatory treatment with OXY plus cisplatin caused an increase in the sub-G1 population. Previous studies have also shown that OXY induces cell cycle arrest in different ways. For example, it induced the accumulation of sub-G1 cells in MDA-MB-231 breast cancer cells [28], while in human lung squamous NCI-H520 cells, it affected cell cycle arrest in the S phase [49]. These findings suggest that OXY’s effects on cell cycle arrest induction may vary depending on the cell type.

Regarding the cell viability study, the results suggest that OXY increased the sensitivity of the cancer cells to chemotherapy. The potential mechanism of OXY may involve the inhibition of cell survival signaling and the production of anti-apoptotic proteins. Thus, we further investigated the levels of anti-apoptotic proteins, including Mcl-1, Bcl-xL, and XIAP. The data demonstrated that EGF stimulation significantly increased the quantity of anti-apoptotic proteins. Conversely, pre-treatment with OXY mitigated these effects. These results are consistent with those of previous research conducted in osteosarcoma and cervical cancer cells [29,34]. The role of these anti-apoptotic proteins is to inhibit the activation of pro-apoptotic proteins, including preventing the recruitment of BAK and BAX to the mitochondrial membrane surface or directly blocking the activation of caspase-9, thereby resisting the induction of cell apoptosis. Many cancers typically carry mutations in survival signaling pathways, resulting in elevated levels of anti-apoptotic proteins that protect cancer cells from immune responses and chemotherapy treatments. Therefore, combining OXY with cisplatin, which requires the activation of pro-apoptotic cells to induce cell death, enhances the efficacy of apoptosis induction. Our data clearly demonstrate a correlation between the production of anti-apoptotic proteins and EGF stimulation. Subsequently, we further investigated the effects of OXY on modulating the signal transduction pathways in response to EGF induction. The Western blot analysis showed that OXY inhibited EGFR phosphorylation in SKOV3 cells, but not in TOV21G cells, without disrupting EGFR degradation. Moreover, OXY reduced the phosphorylation of AKT and PDK1 downstream of EGFR, while its effect on phosphorylated mTOR remained unchanged. Interestingly, OXY specifically inhibited the PI3K/AKT/mTOR pathway without affecting the MAPK pathway. The inhibition of phosphorylated AKT by OXY is consistent with the reports of previous studies showing its effect on reducing IL-1β release in human microglia HMC3 cells, at least in part through suppressing AKT phosphorylation [32]. Additionally, in one study, it was found that OXY inhibited phosphorylated AKT in HeLa cervical cancer cells, thus inhibiting cancer cell proliferation and migration [50]. However, the exact mechanism of action that would explain how OXY specifically inhibits kinases and substrates in certain signaling pathways is not yet known. Based on the fact that all kinases naturally phosphorylate substrates in the context of specific sequence motifs, structural biology, as well as designing synthetic signaling pathways to create a functional protein kinase–substrate interaction [50], may help in identifying the specific target positions of OXY on the kinases. In this study, we observed differences between SKOV3 and TOV21G cells in their response to OXY. TOV21G cells harbored mutations in the PI3K and PTEN genes, resulting in a constitutive activation of the PI3K/AKT/mTOR pathway even in the absence of EGF stimulation [21]. This was evident based on the relatively high basal level of phosphorylated AKT in the untreated TOV21G cells, which is consistent with our other findings regarding these specific ovarian cancer cells expressing high amount of anti-apoptotic proteins (Mcl-1, XIAP, and Bcl-xL). Moreover, this characteristic limited the maximum level of exogenous EGF stimulation, causing the TOV21G cells to respond less to EGF than the SKOV3 cells. The constitutive activation of the PI3K/AKT/mTOR pathway reduced the efficacy of OXY in cancer cells, rendering the TOV21G cells resistant to its effects on cell apoptosis induction. Nonetheless, OXY remains a potential candidate for use in combination with other chemotherapies. SKOV3 cells, which lack TP53, a crucial protein in regulating cell apoptosis, exhibit a certain degree of resistance to cisplatin treatment. Co-administration of OXY with cisplatin sensitized ovarian cancer cells, enhancing their response to chemotherapy and promoting apoptosis induction. Therefore, oxyresveratrol may be a good active compound, in addition to its well-known derivative, resveratrol, for use in combination with other agents for treating ovarian cancer.

## 5. Conclusions

**Figure 8:**
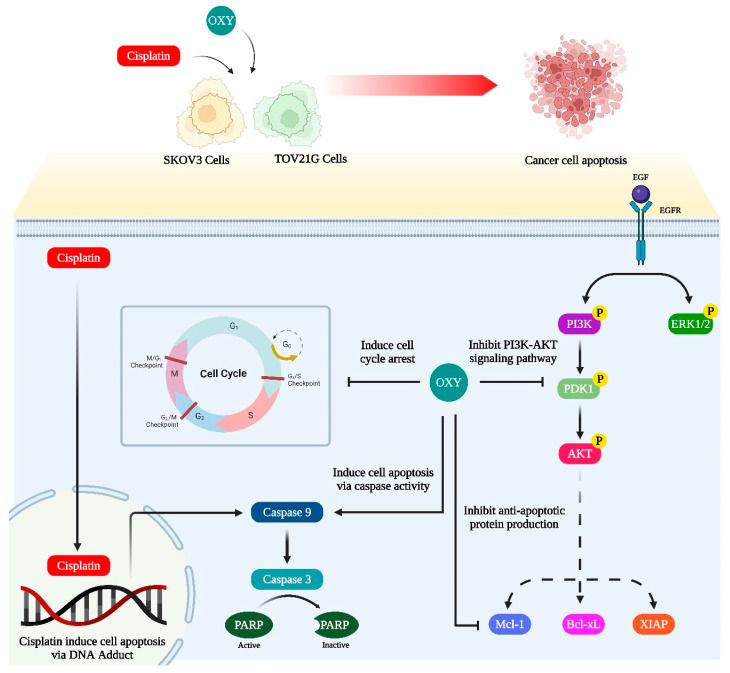
Schematic illustration proposing the mechanism of oxyresveratrol in enhancing the effects of cisplatin through inducing cell cycle arrest, inhibiting the PI3K/AKT signaling pathway, inhibiting the production of anti-apoptotic proteins, and inducing cell apoptosis in ovarian cancer cells. This illustration was created through BioRender.com (accessed on 22 July 2024).

As depicted in the schematic illustration below (Figure 8), OXY is a natural compound with potential anti-cancer effects. However, its exact molecular mechanisms and efficacy, particularly across different types of cancer cells, require further investigation. The current study contributes additional evidence supporting OXY’s ability to reduce cancer cell viability, induce apoptosis, arrest cell cycle progression, and enhance cancer cells’ sensitivity to chemotherapy. The potential molecular mechanisms behind its effects may involve the inhibition of the PI3K/AKT/mTOR pathway, leading to a reduced production of anti-apoptotic proteins. These effects underscore the potential of combining OXY with conventional chemotherapy to improve treatment efficacy. Importantly, this study highlights variations in OXY’s efficacy across different ovarian cancer cell types and suggests caution in its use for cancers with constitutive activation of the survival signaling pathways. The data presented in this work serve as foundational knowledge for further understanding and developing novel anti-cancer medications, potentially guiding future clinical applications.

## Data Availability

All data, tables and figures are original and are available in this article.

## Supplementary Materials

The following supporting information can be downloaded at https://www.mdpi.com/article/10.3390/biom14091140/s1: Figure S1: The combination index of OXY and cisplatin co-treatment in relation to cell viability at each fraction-affected concentration in SKOV3 and TOV21G cells. Original images of Figure 2C, Figure 4, Figure 5, Figure 6 and Figure 7 can be found in Supplementary Materials.
