# Supplementary material for: Oxyresveratrol Enhances the Anti-Cancer Effect of Cisplatin against Epithelial Ovarian Cancer Cells through Suppressing the Activation of Protein Kinase B (AKT)
Source: Biomolecules. 2024 Sep 9;14(9):1140. doi: 10.3390/biom14091140 (PMC11430010; doi:10.3390/biom14091140)

SKOV3

Cell apoptosis experiment

3 repeated results represented Fig 2 (C)

Repeat 1 Repeat 2 Repeat 3

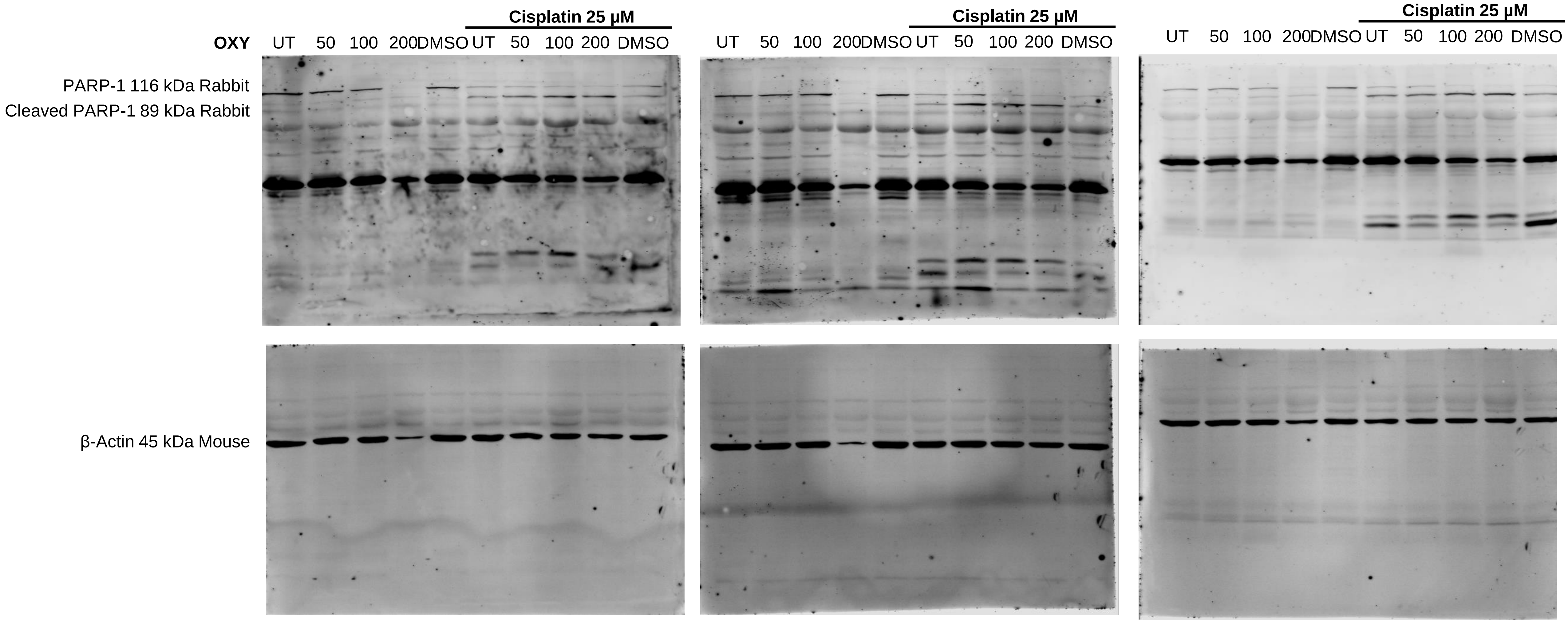

SKOV3

Cell apoptosis experiment

3 repeated results represented Fig 2 (C)

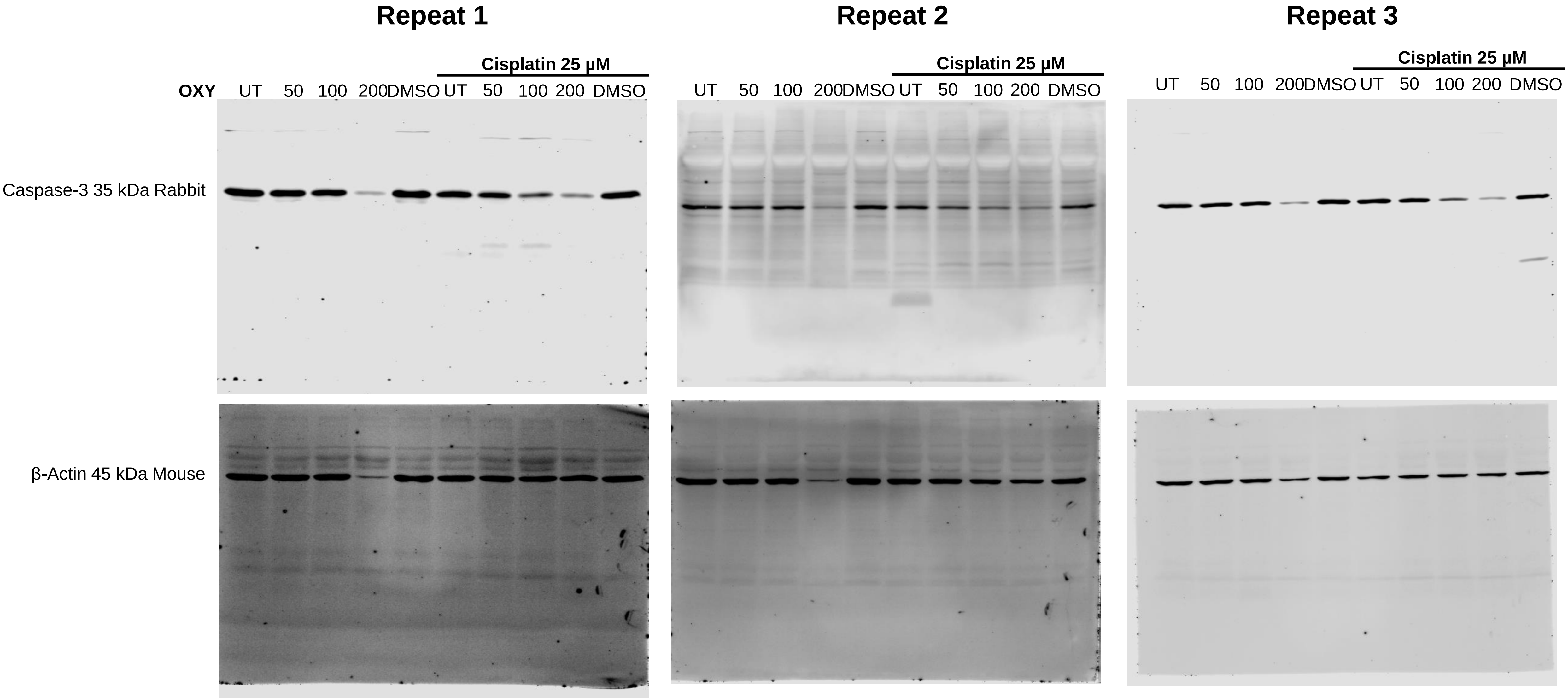

SKOV3

Cell apoptosis experiment

3 repeated results represented Fig 2 (C)

Repeat 1 | Repeat 2 | Repeat 3

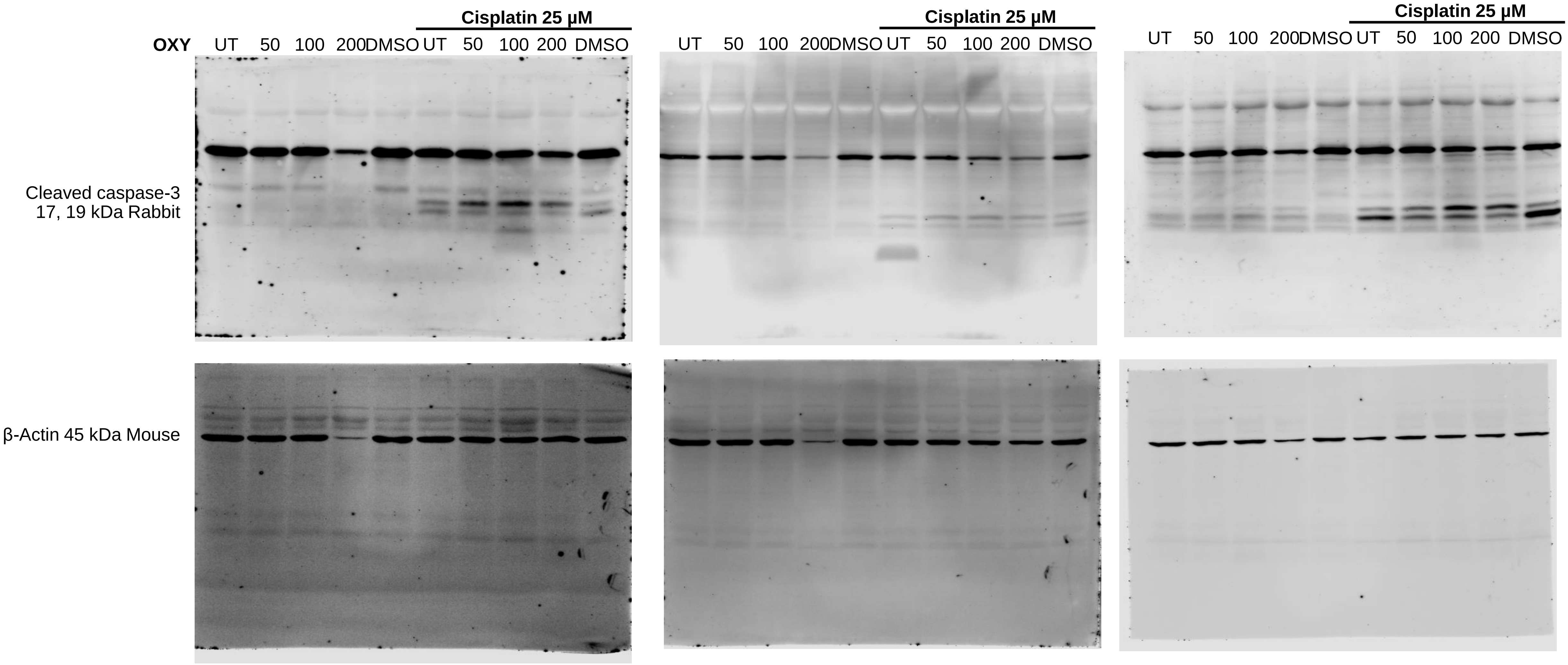

SKOV3

Cell apoptosis experiment

3 repeated results represented Fig 2 (C)

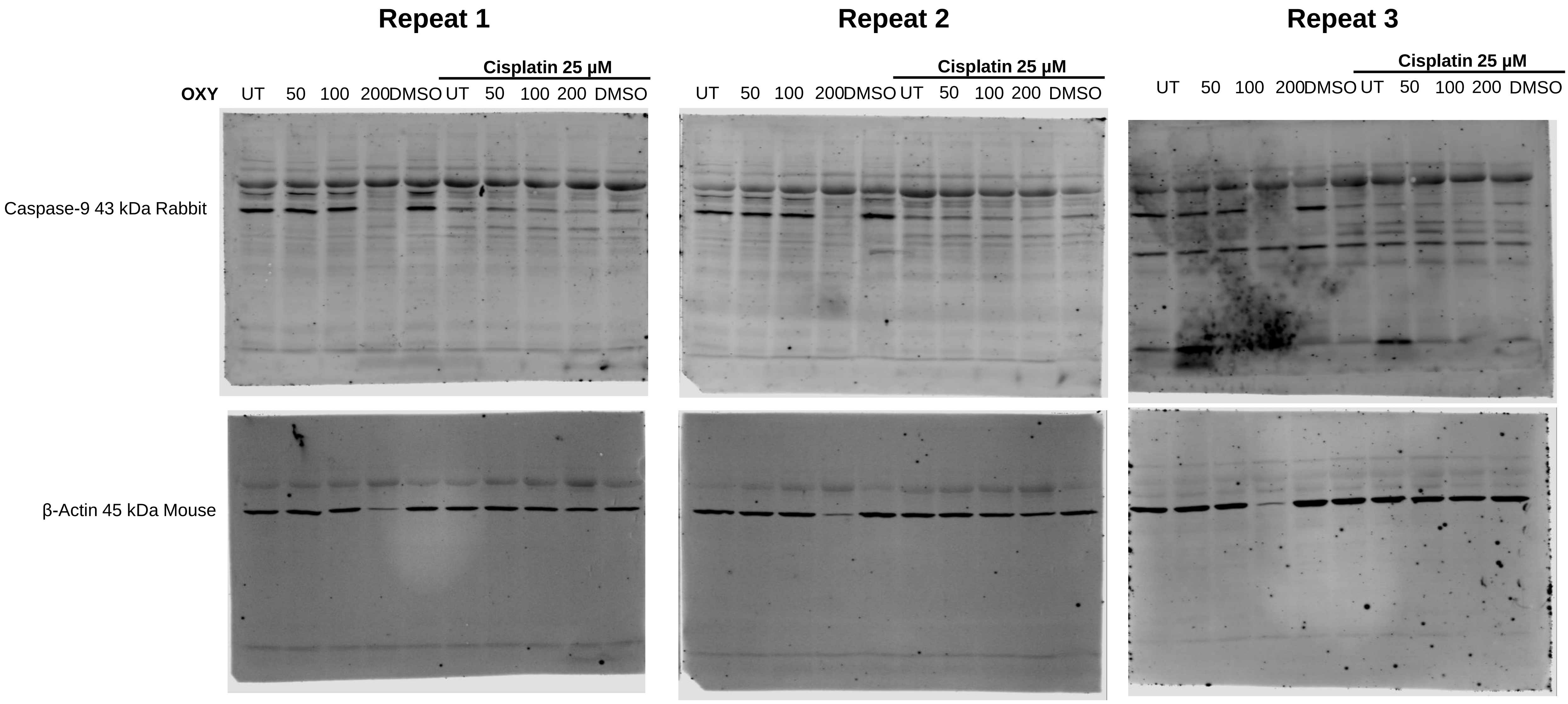

SKOV3

Cell apoptosis experiment

3 repeated results represented Fig 2 (C)

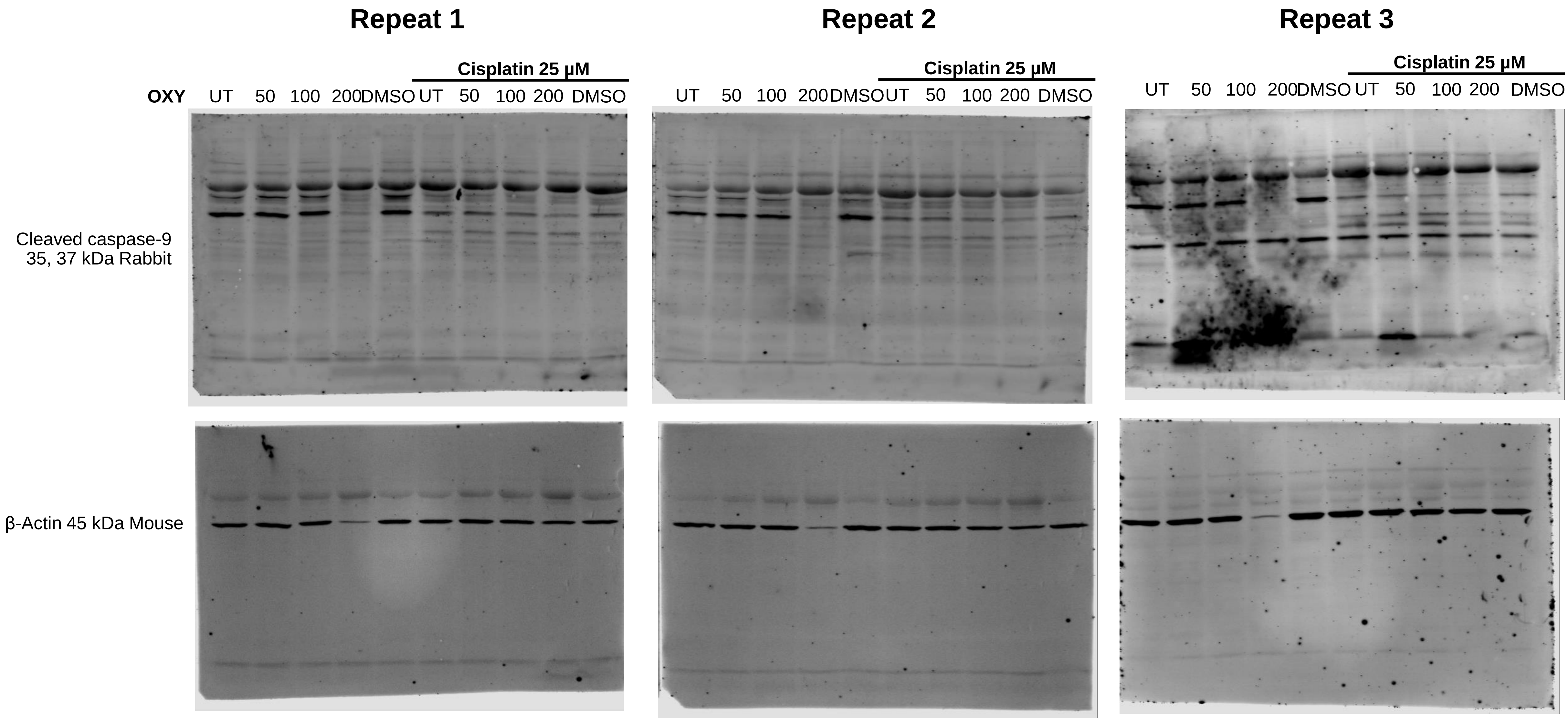

TOV21G

Cell apoptosis experiment

3 repeated results represented Fig 2 (C)

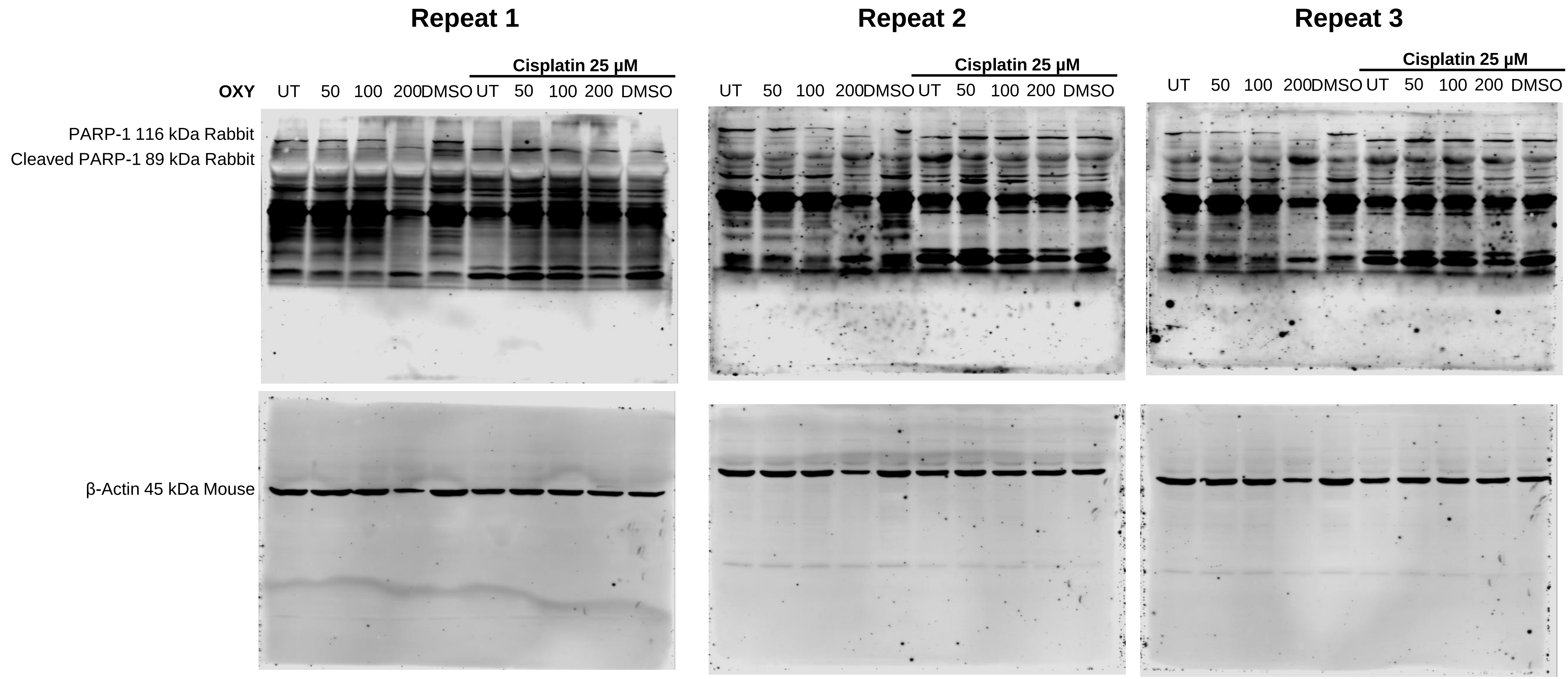

TOV21G

Cell apoptosis experiment

3 repeated results represented Fig 2 (C)

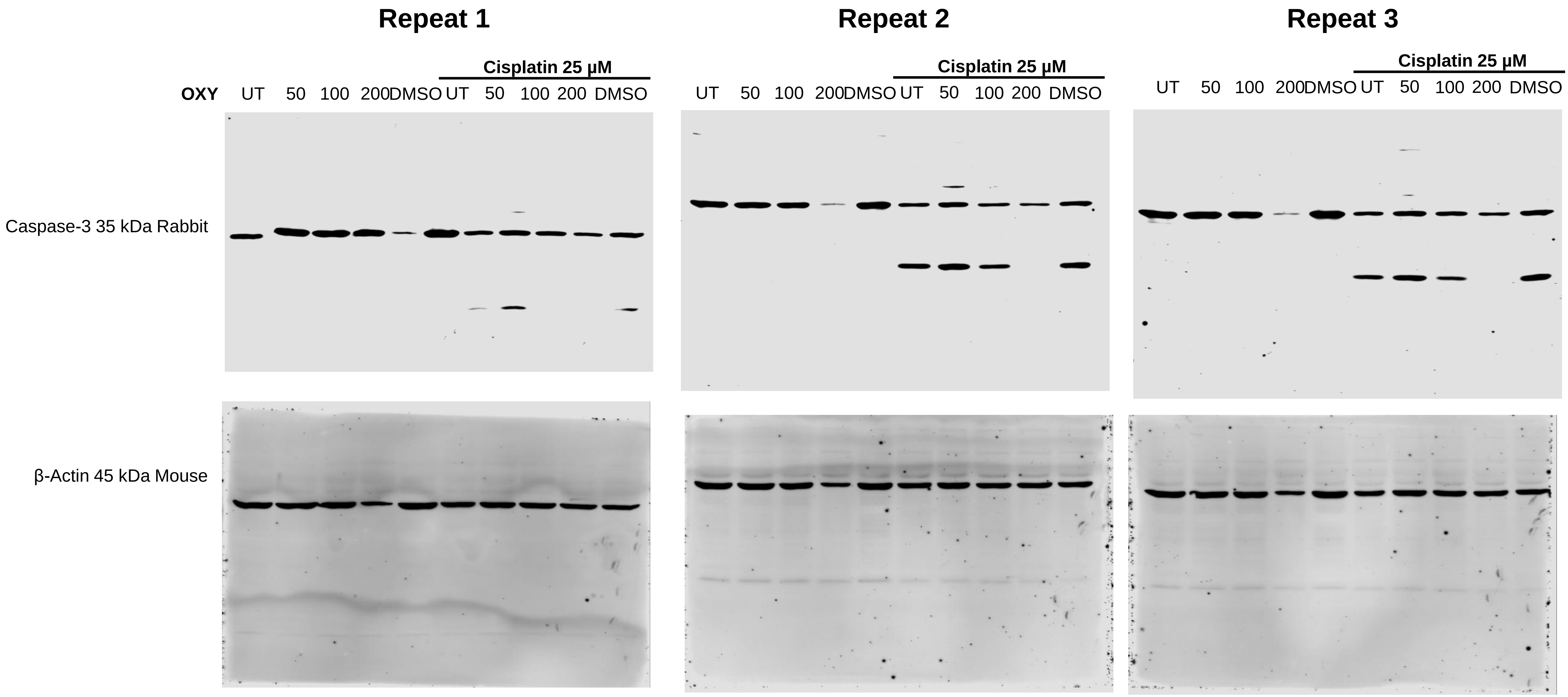

TOV21G

Cell apoptosis experiment

3 repeated results represented Fig 2 (C)

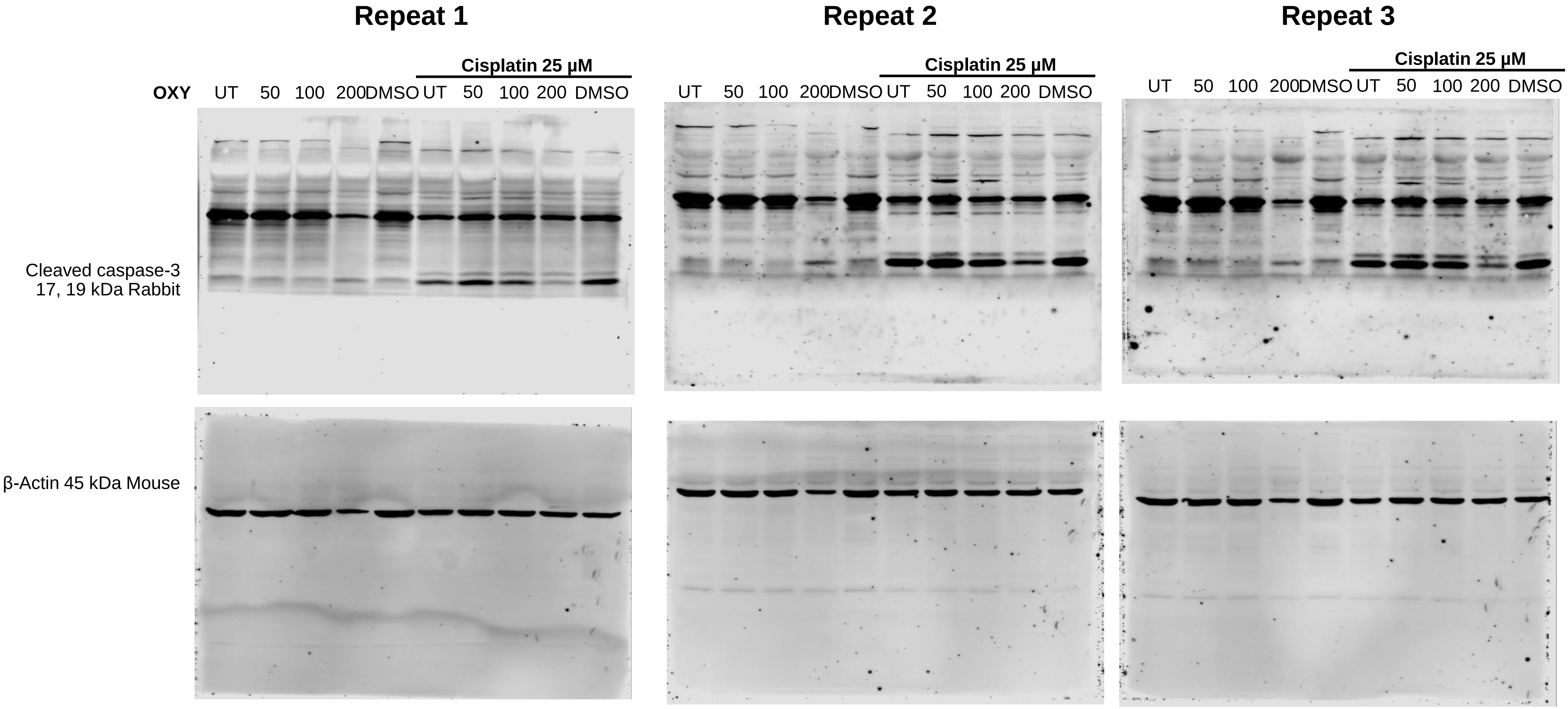

TOV21G

Cell apoptosis experiment

3 repeated results represented Fig 2 (C)

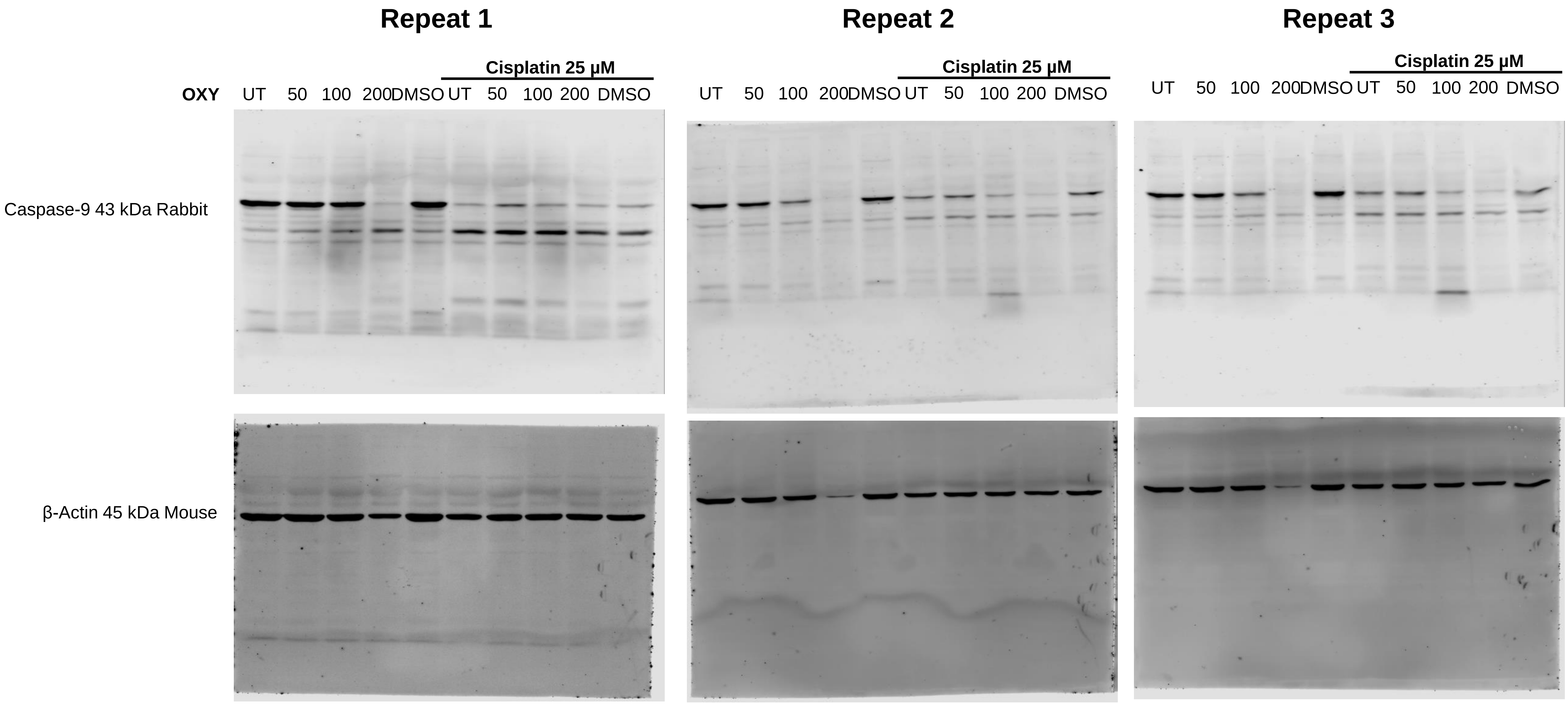

TOV21G

Cell apoptosis experiment

3 repeated results represented Fig 2 (C)

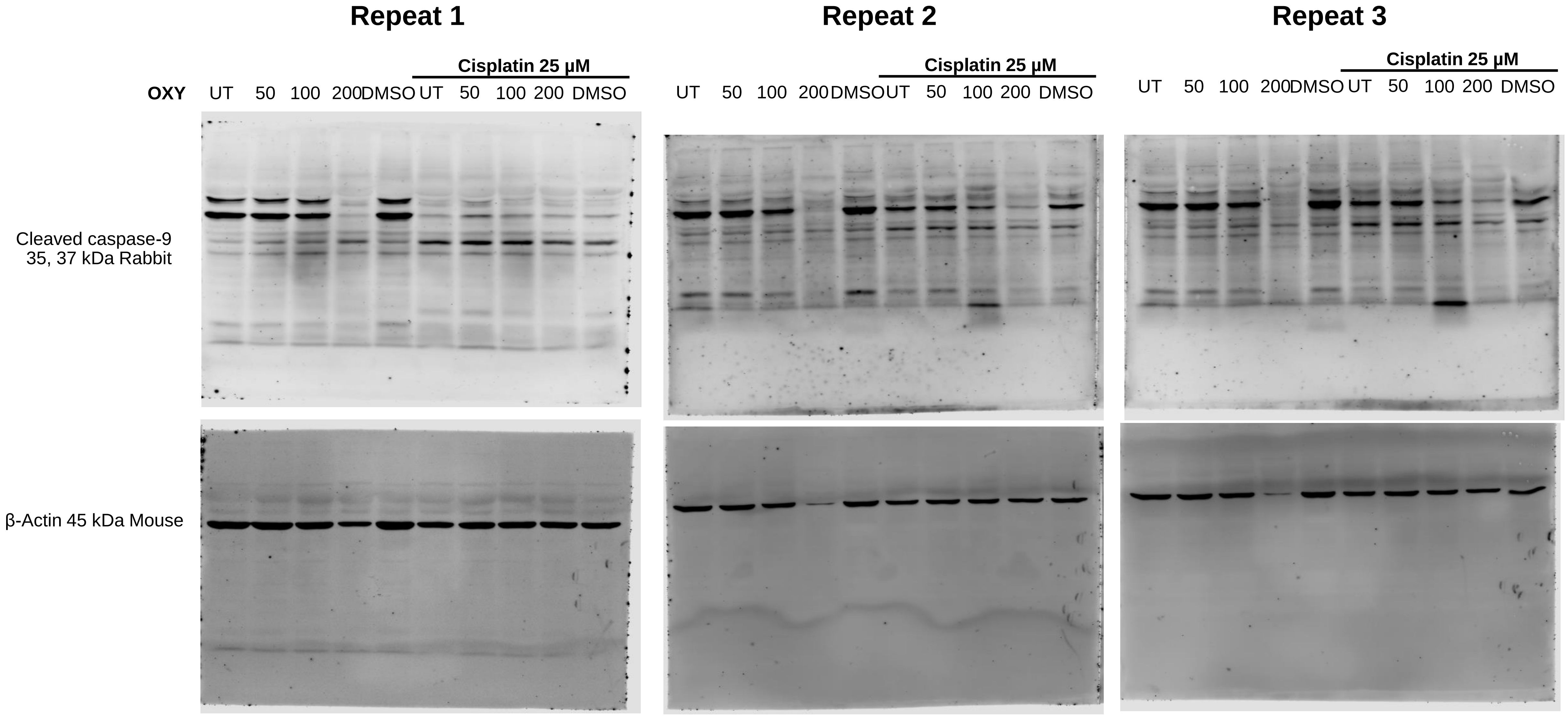

SKOV3

Cell apoptosis experiment

3 repeated results represented Fig 4

	Repeat 1	Repeat 2	Repeat 3
	**OXY** UT EGF 50 100 200	**OXY** UT EGF 50 100 200	**OXY** UT EGF 50 100 200

Mcl-1 35, 40 kDa Rabbit

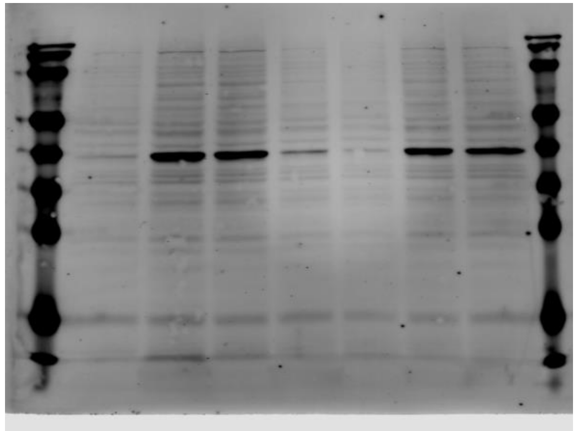

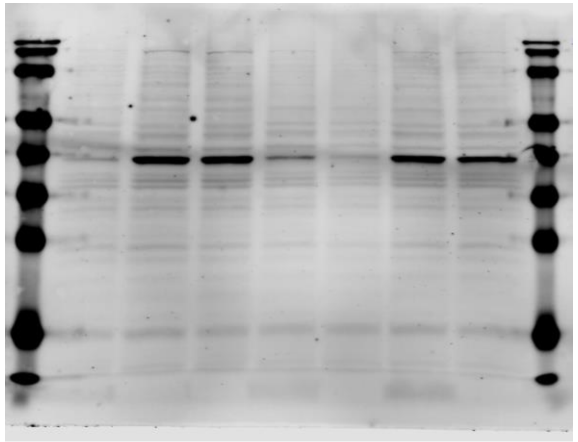

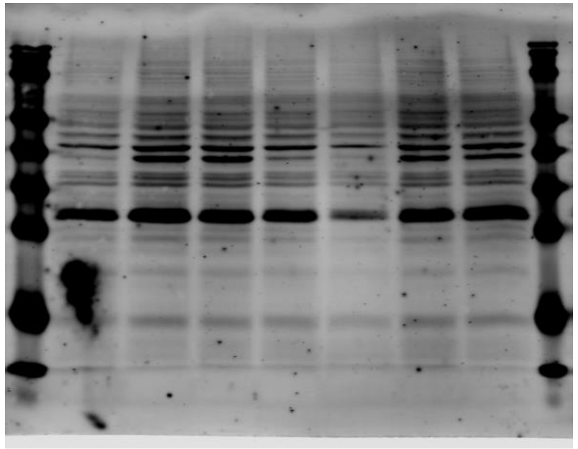

β-Actin 45 kDa Mouse

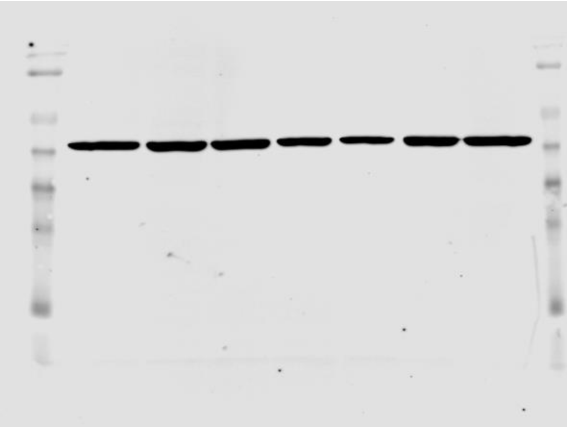

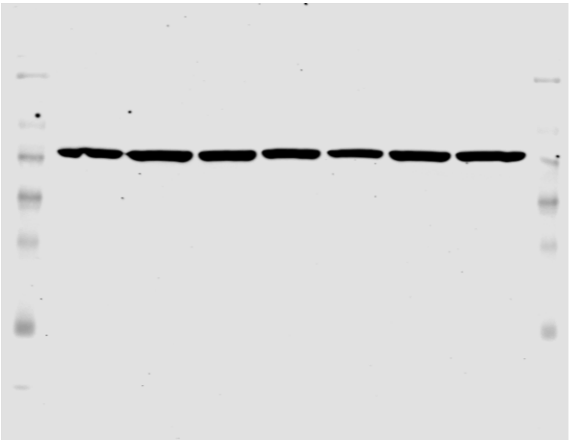

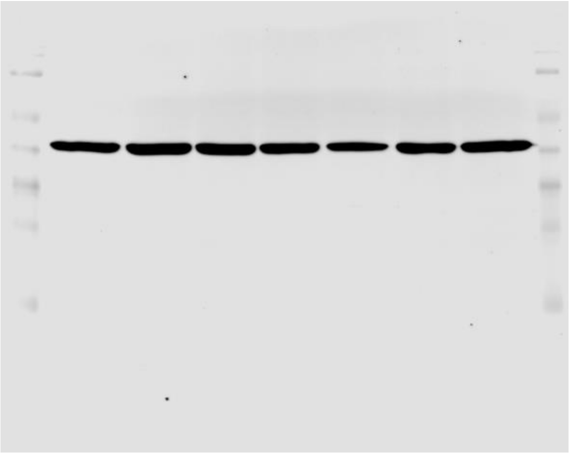

SKOV3

Cell apoptosis experiment

3 repeated results represented Fig 4

Repeat 1 | Repeat 2 | Repeat 3

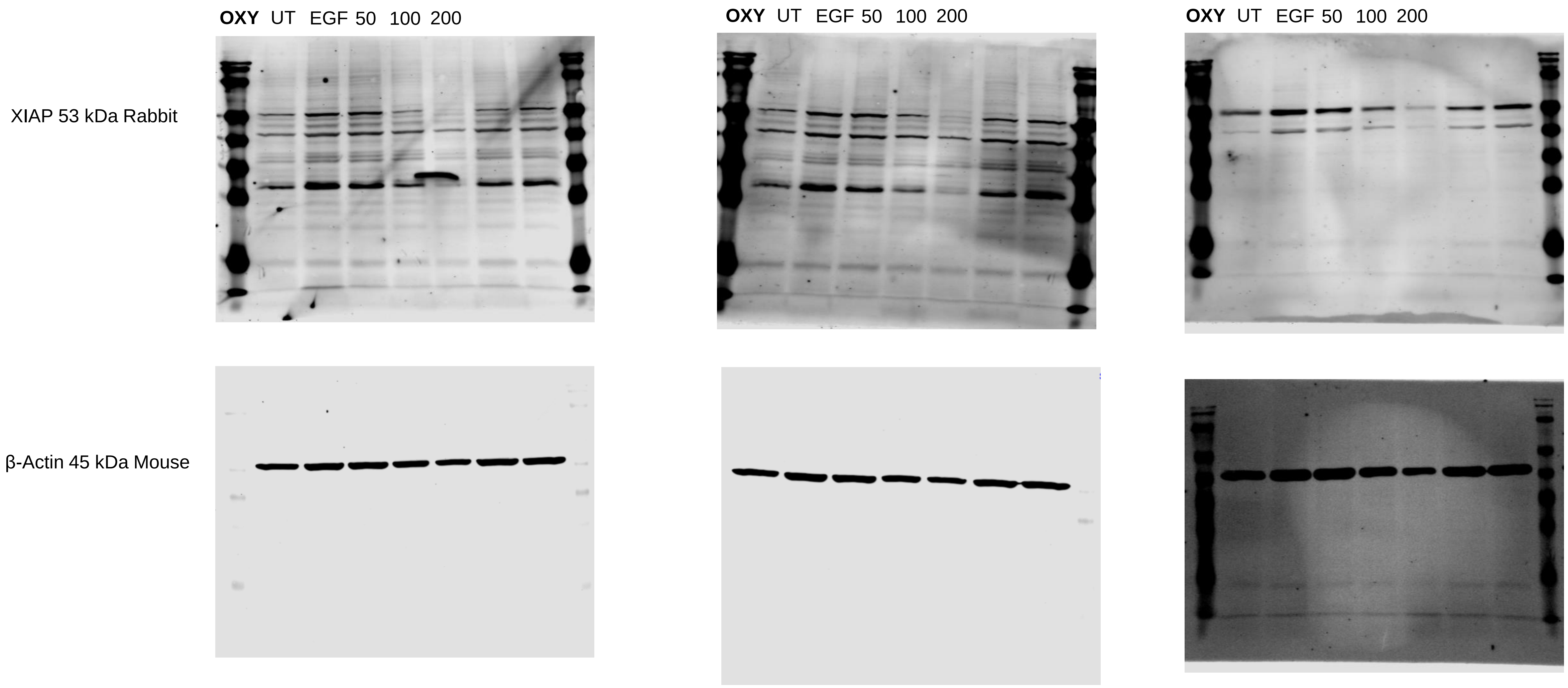

SKOV3

Cell apoptosis experiment

3 repeated results represented Fig 4

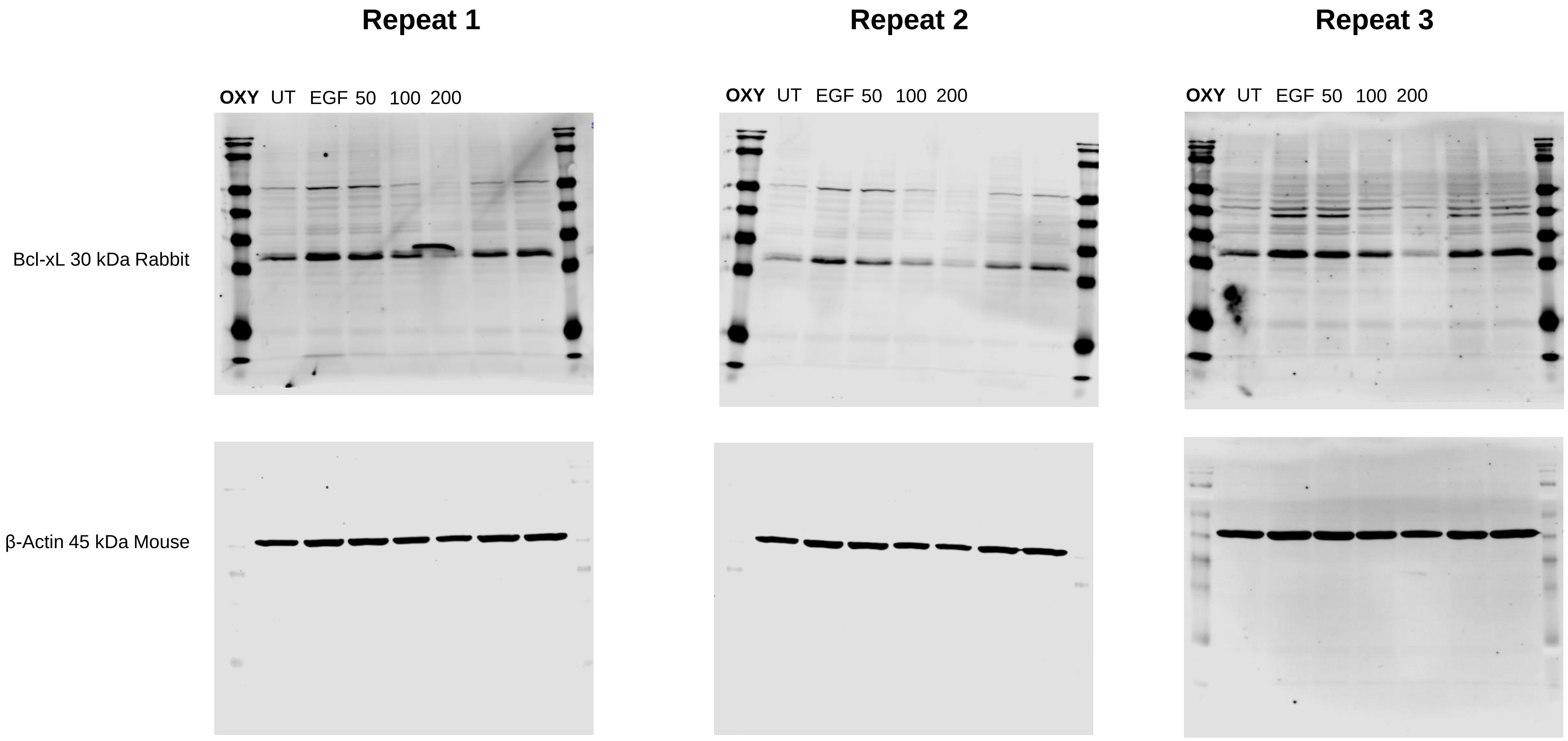

TOV21G

Cell apoptosis experiment

3 repeated results represented Fig 4

Repeat 1

OXY UT EGF 50 100 200

Mcl-1 35, 40 kDa Rabbit

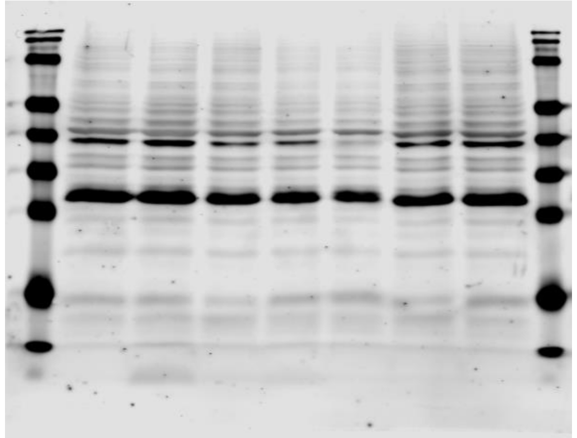

β-Actin 45 kDa Mouse

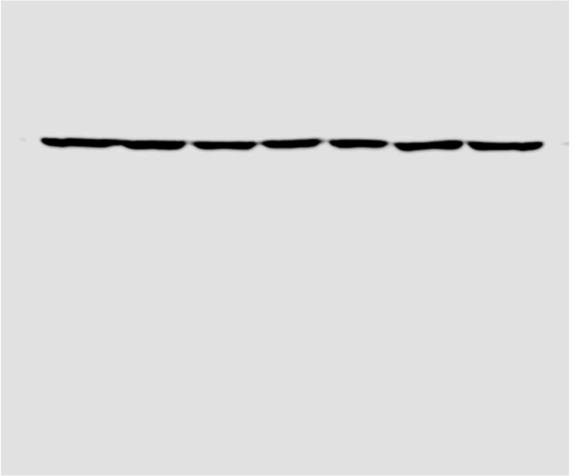

Repeat 2

OXY UT EGF 50 100 200

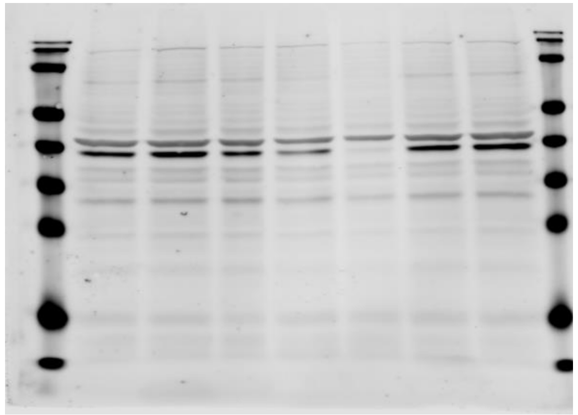

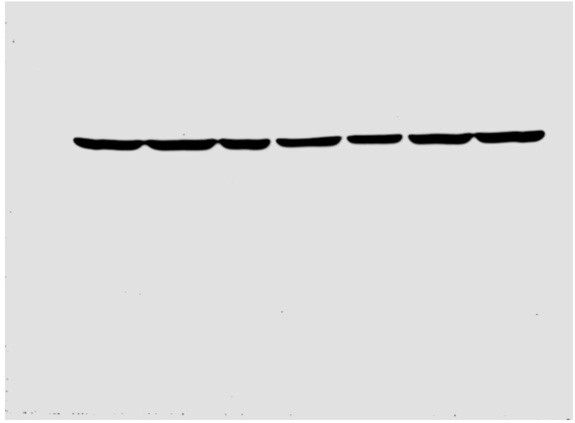

Repeat 3

OXY UT EGF 50 100 200

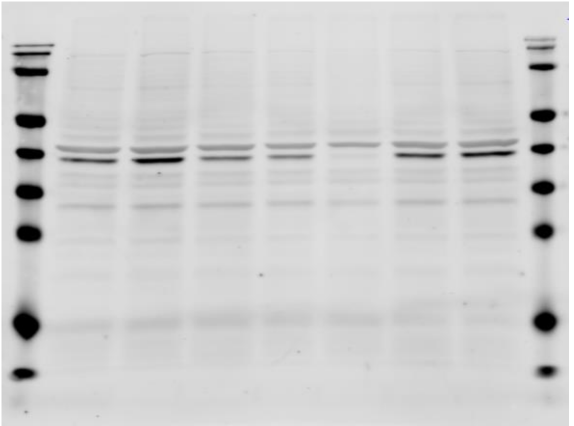

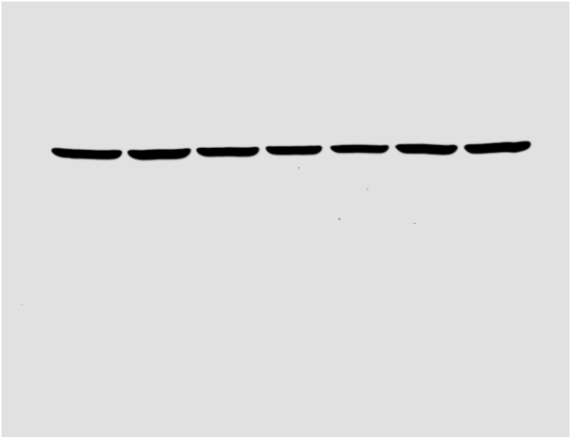

TOV21G

Cell apoptosis experiment

3 repeated results represented Fig 4

Repeat 1 | Repeat 2 | Repeat 3

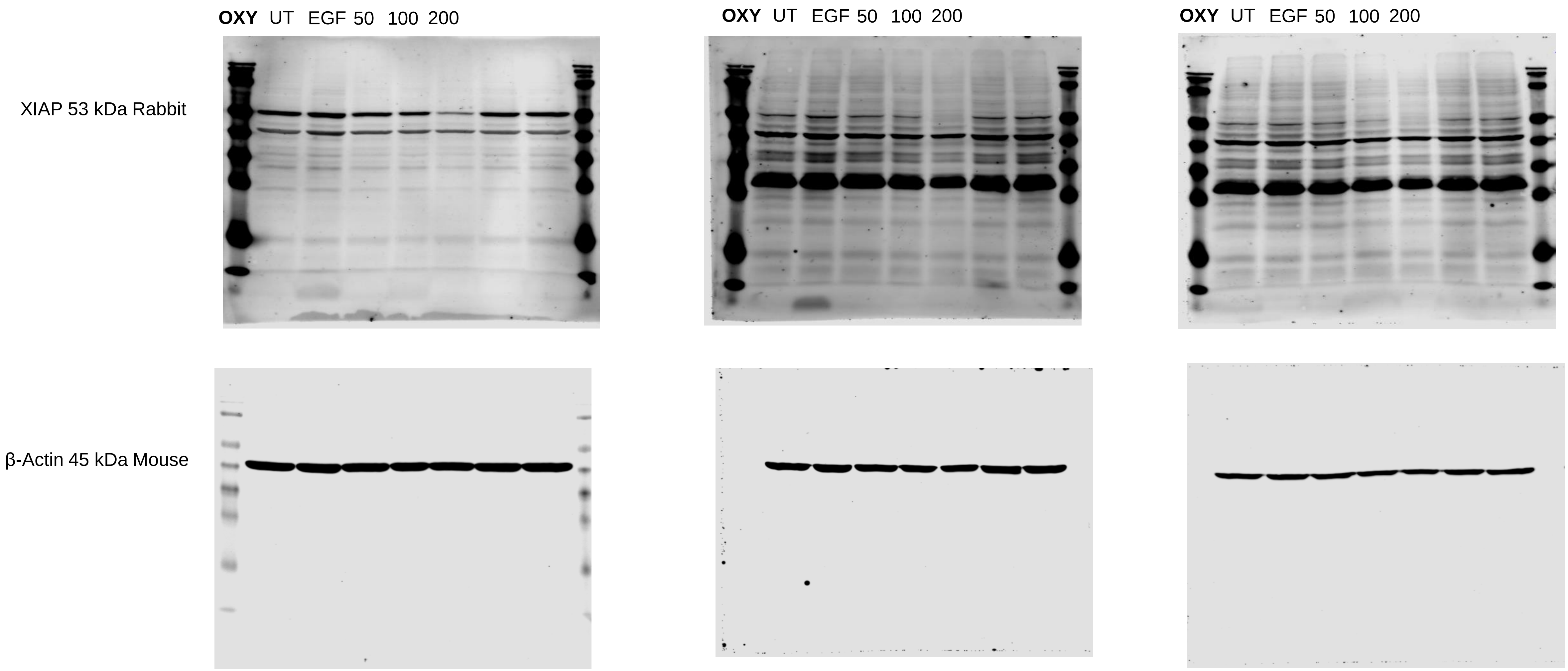

TOV21G

Cell apoptosis experiment

3 repeated results represented Fig 4

Repeat 1

OXY UT EGF 50 100 200

Bcl-xL 30 kDa Rabbit

β-Actin 45 kDa Mouse

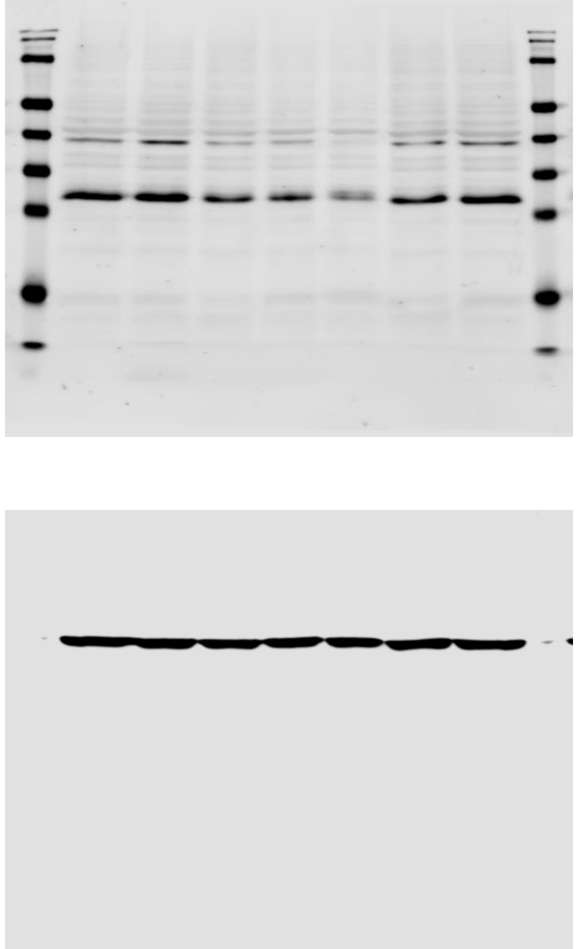

Repeat 2

OXY UT EGF 50 100 200

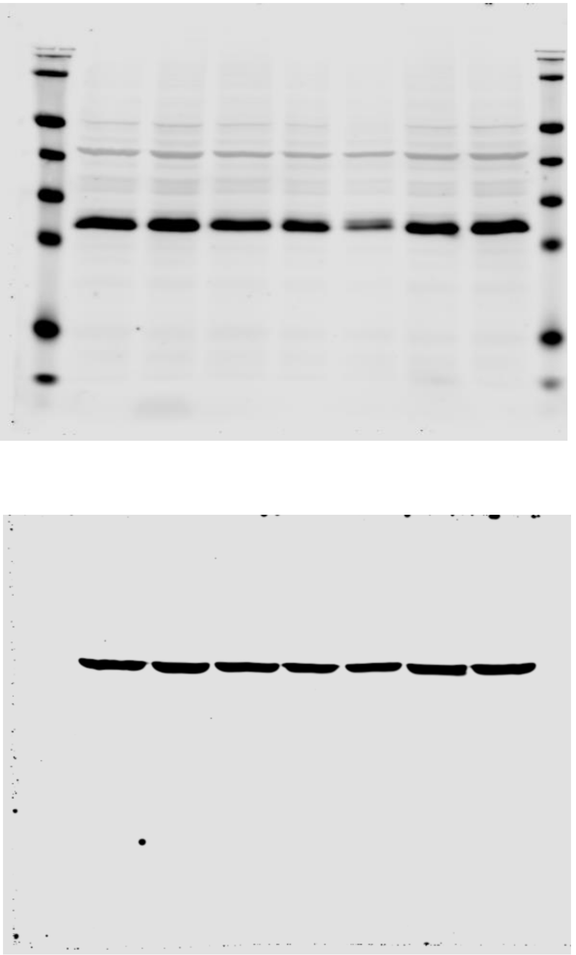

Repeat 3

OXY UT EGF 50 100 200

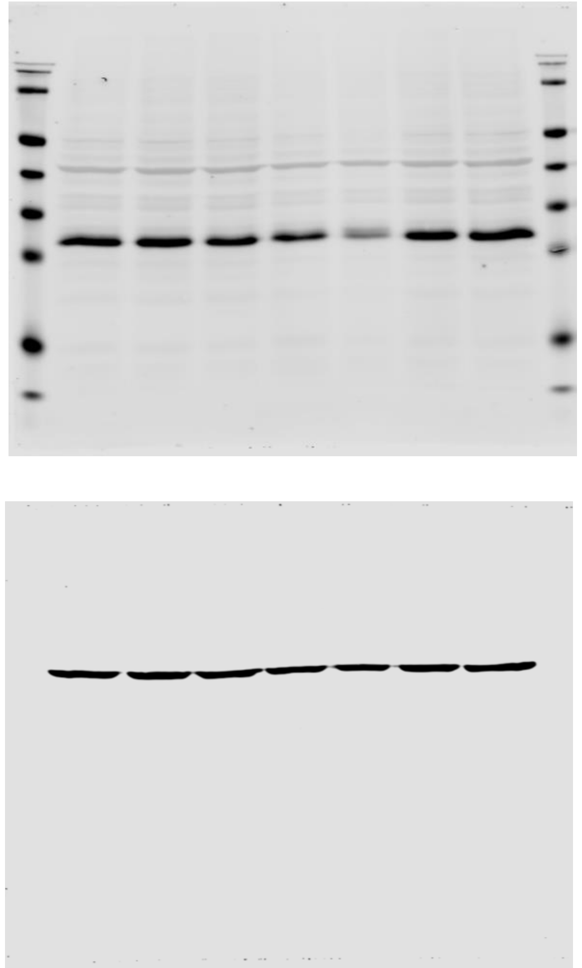

SKOV3

Cell apoptosis experiment

3 repeated results represented Fig 5

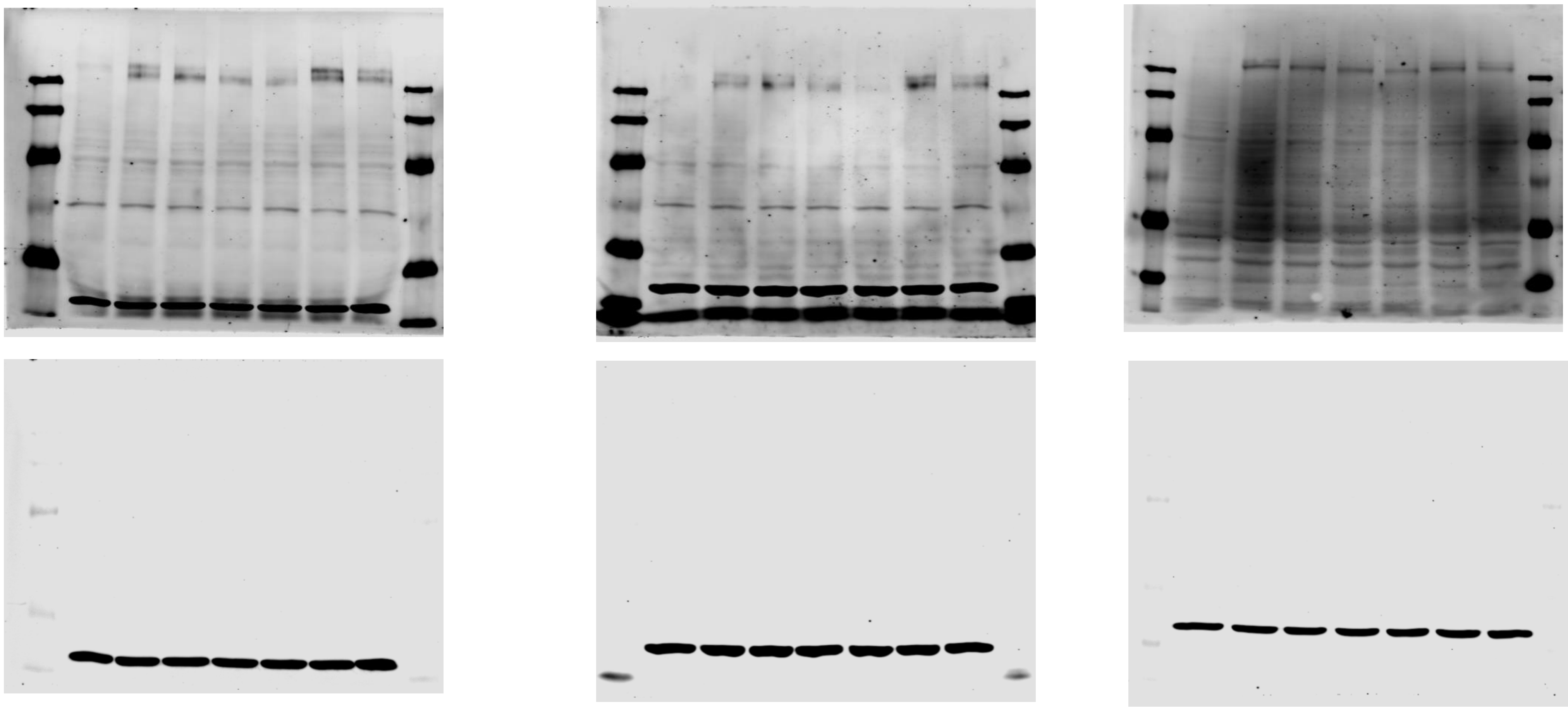

SKOV3

Cell apoptosis experiment

3 repeated results represented Fig 5

Repeat 1 **Repeat 2** **Repeat 3**

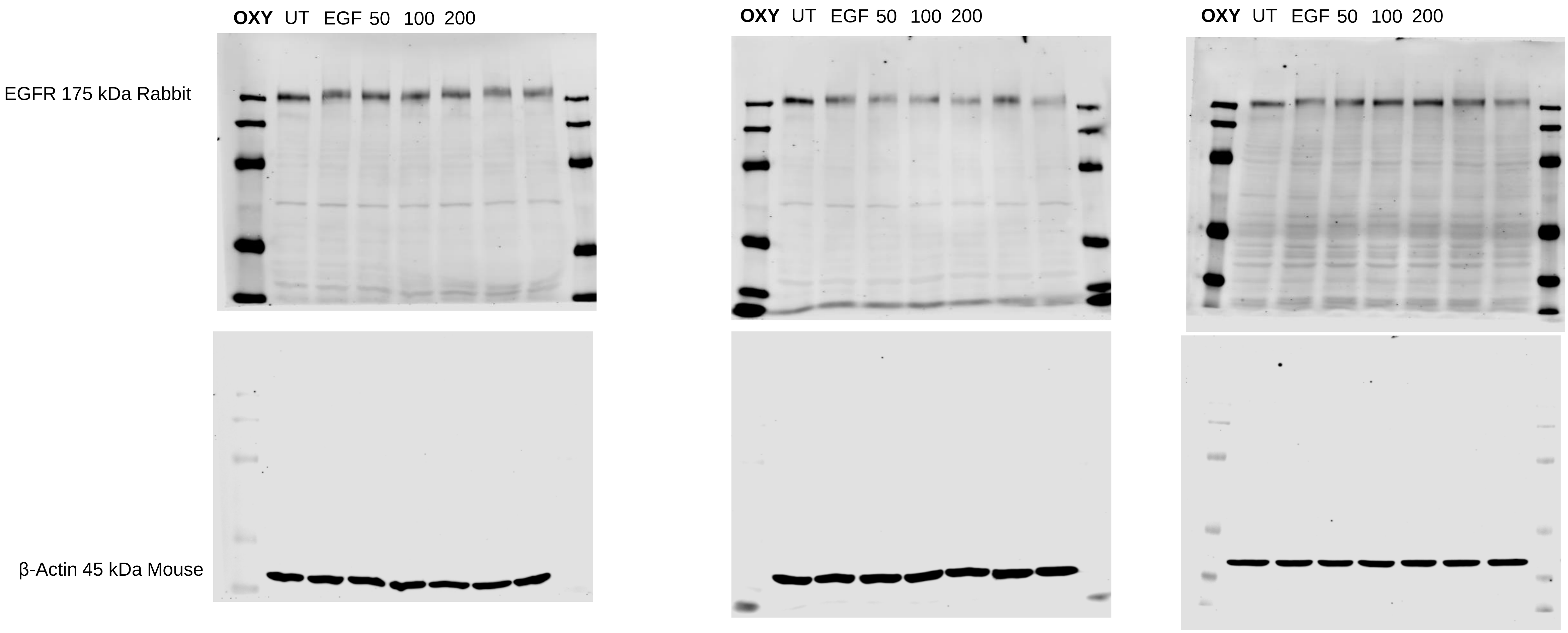

TOV21G

Cell apoptosis experiment

3 repeated results represented Fig 5

Repeat 1

OXY UT EGF 50 100 200

p-EGFR 175 kDa Rabbit

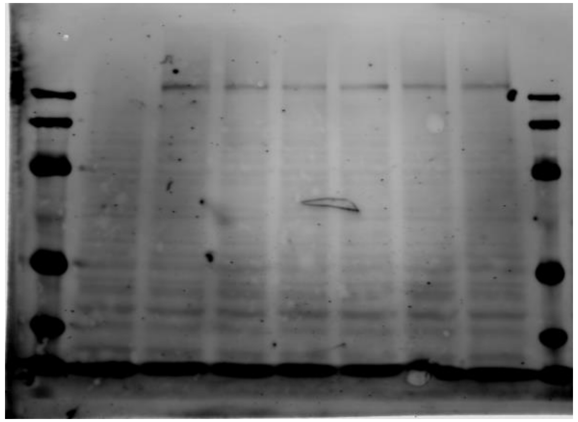

β-Actin 45 kDa Mouse

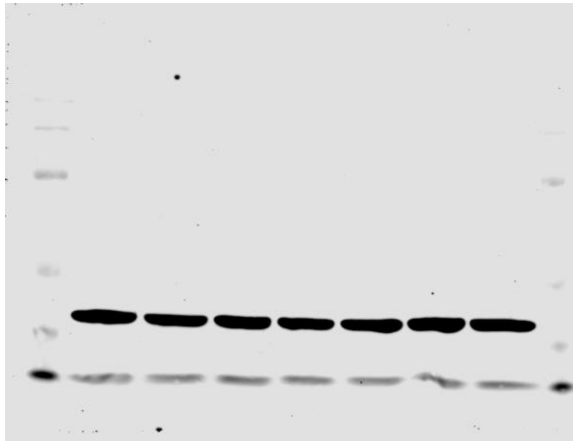

Repeat 2

OXY UT EGF 50 100 200

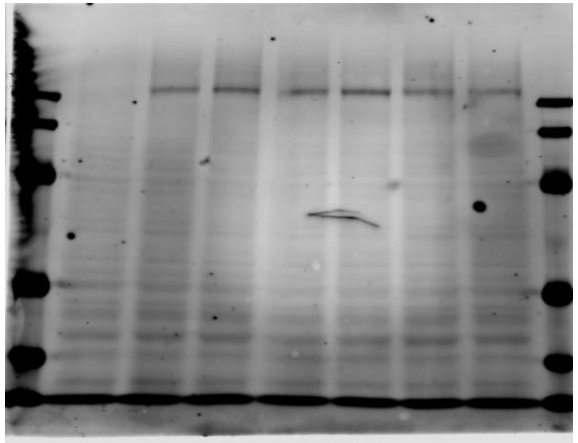

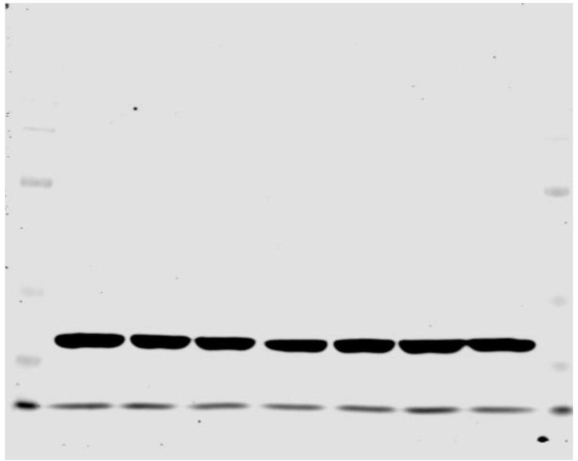

Repeat 3

OXY UT EGF 50 100 200

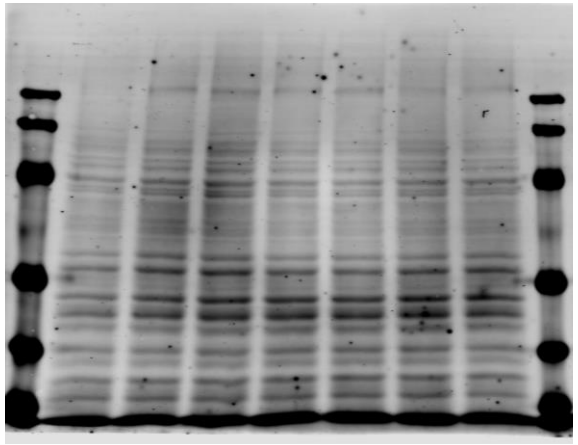

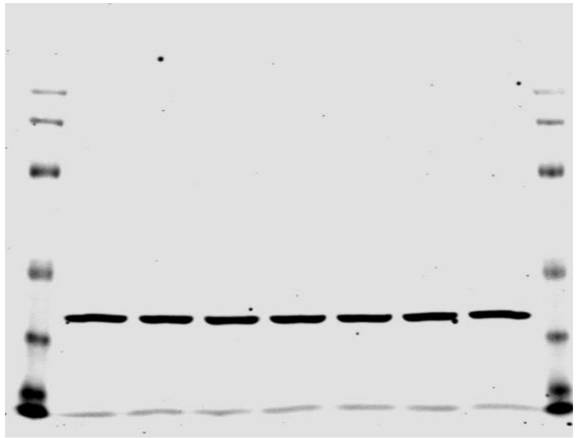

TOV21G

Cell apoptosis experiment

3 repeated results represented Fig 5

Repeat 1 | **Repeat 2** | **Repeat 3**

OXY UT EGF 50 100 200 | **OXY** UT EGF 50 100 200 | **OXY** UT EGF 50 100 200

EGFR 175 kDa Rabbit

β-Actin 45 kDa Mouse

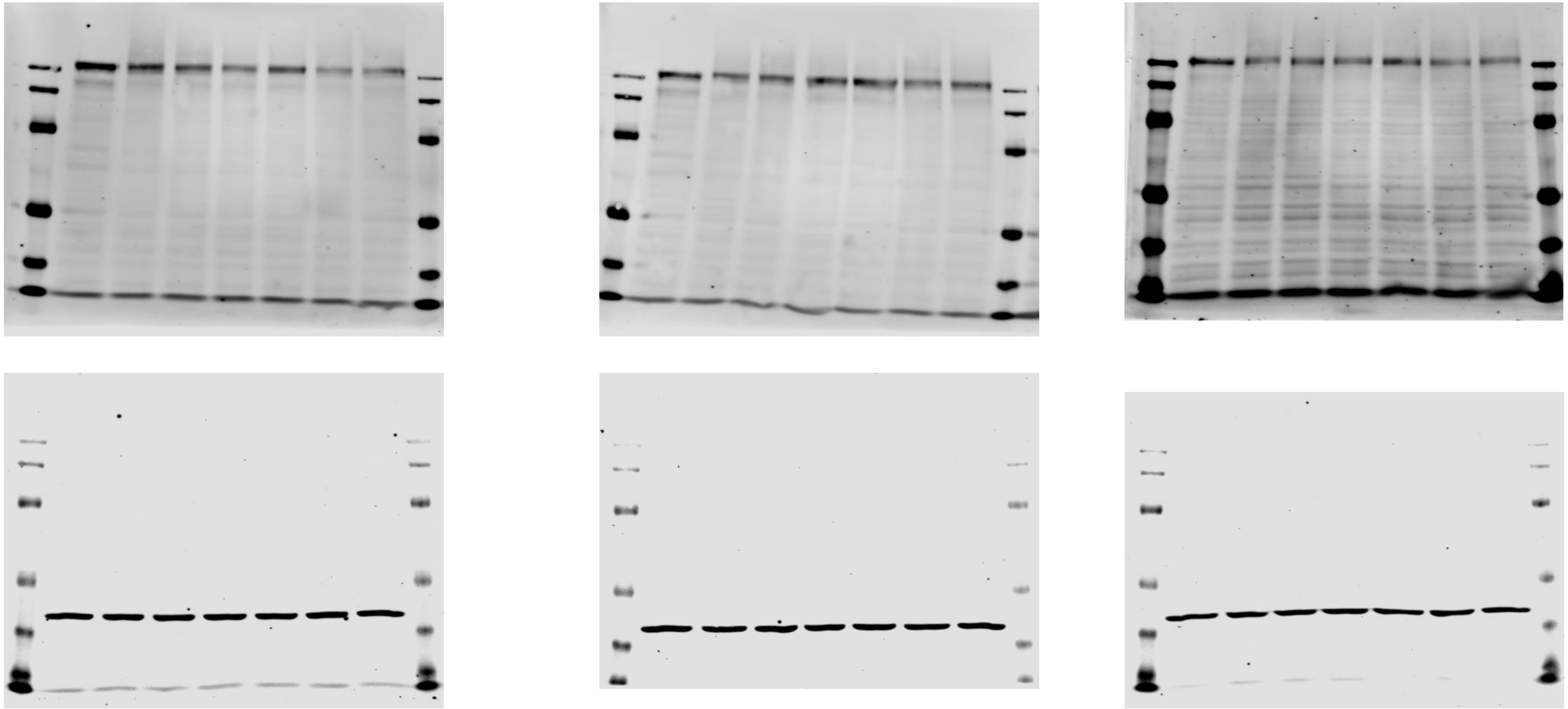

SKOV3

Cell apoptosis experiment

3 repeated results represented Fig 6

Repeat 1 Repeat 2 Repeat 3

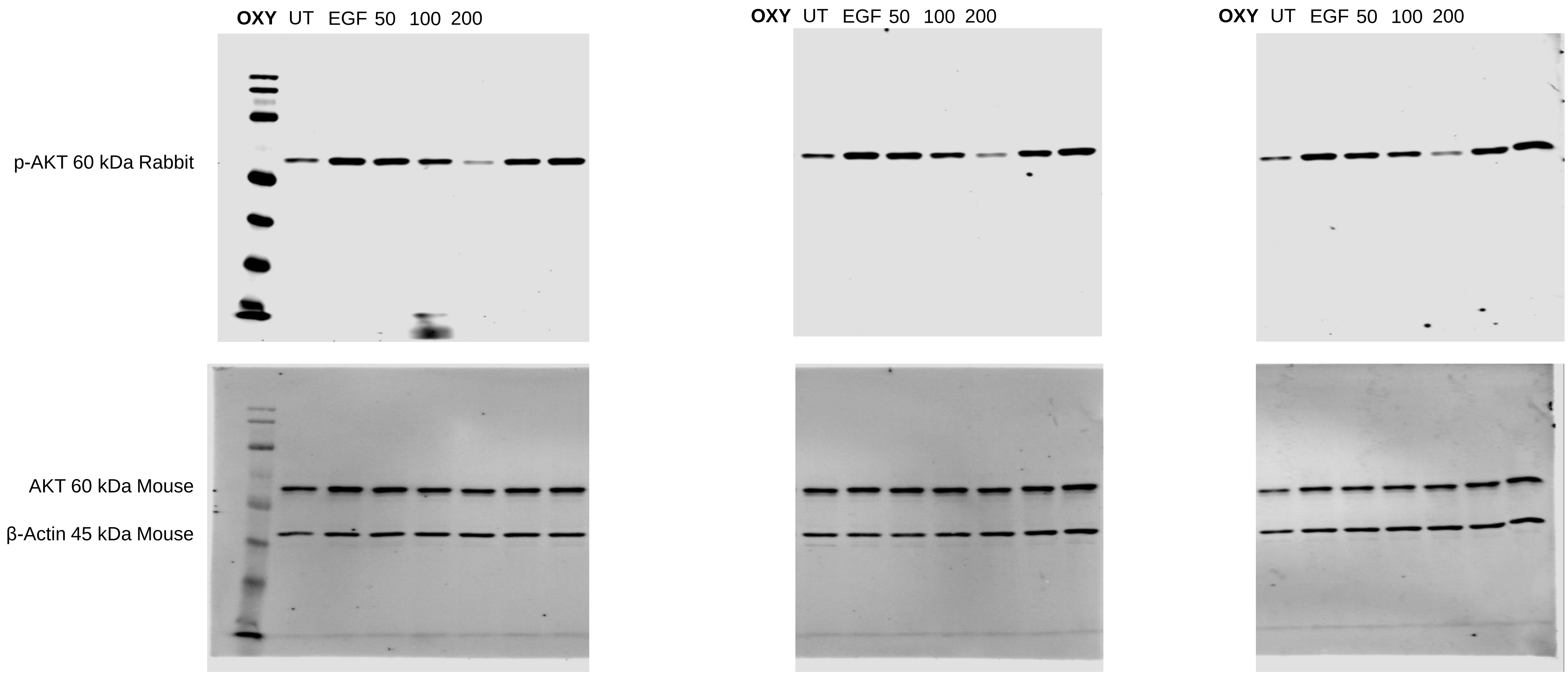

SKOV3

Cell apoptosis experiment

3 repeated results represented Fig 6

Repeat 1

OXY UT EGF 50 100 200

Repeat 2

OXY UT EGF 50 100 200

Repeat 3

OXY UT EGF 50 100 200

p-PDK1 175 kDa Rabbit

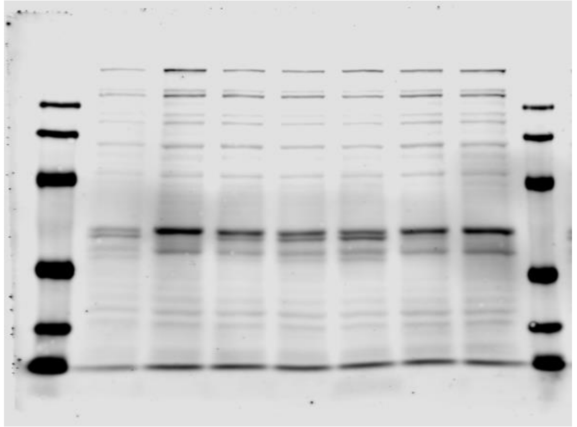

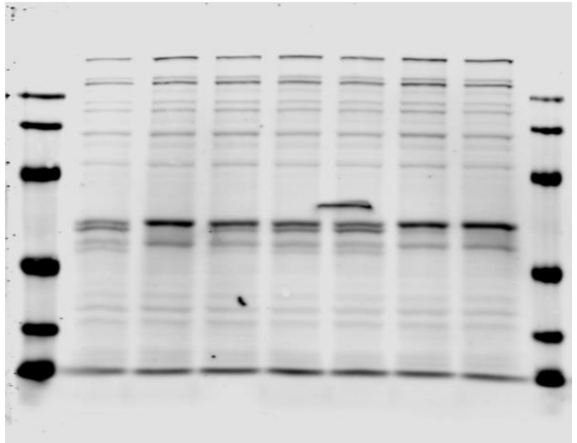

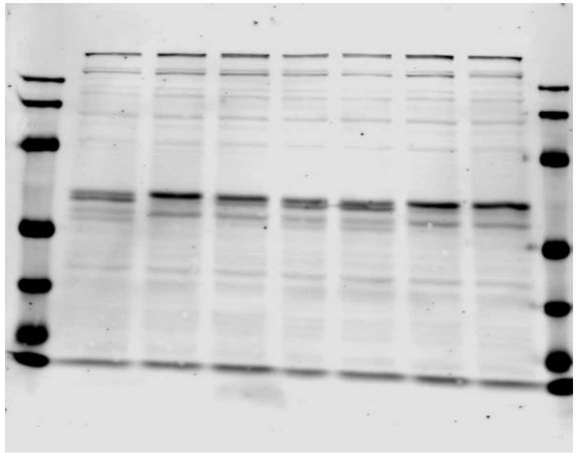

PDK1 175 kDa Rabbit

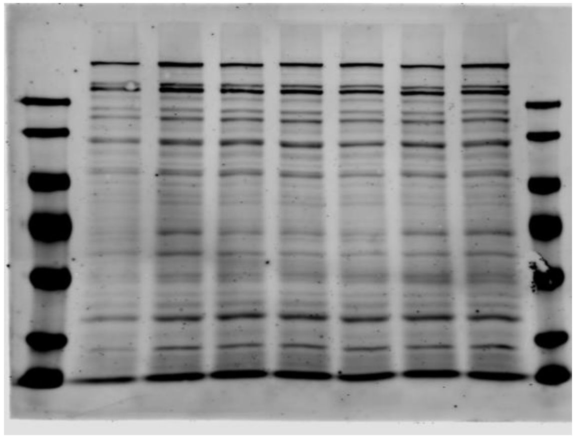

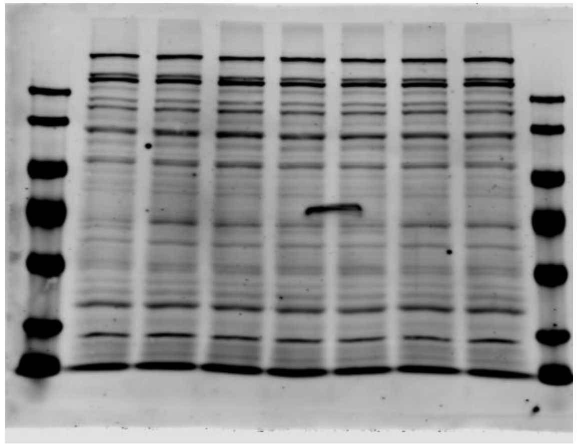

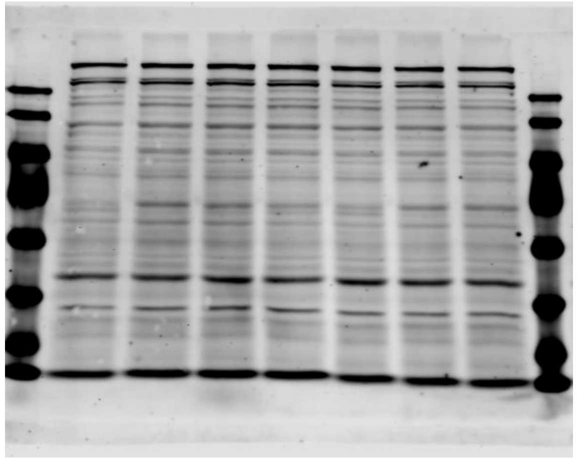

SKOV3

Cell apoptosis experiment

3 repeated results represented Fig 6

Repeat 1

OXY UT EGF 50 100 200

p-mTOR 289 kDa Rabbit

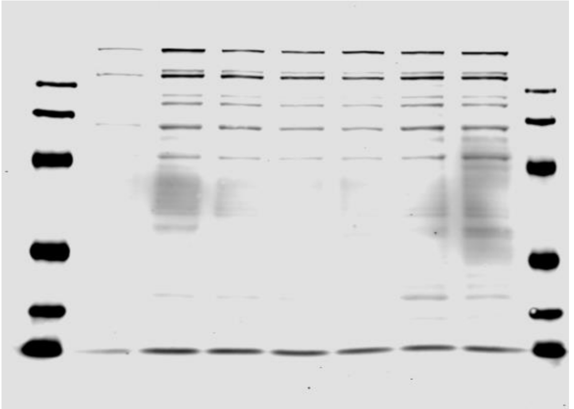

mTOR 289 kDa Rabbit

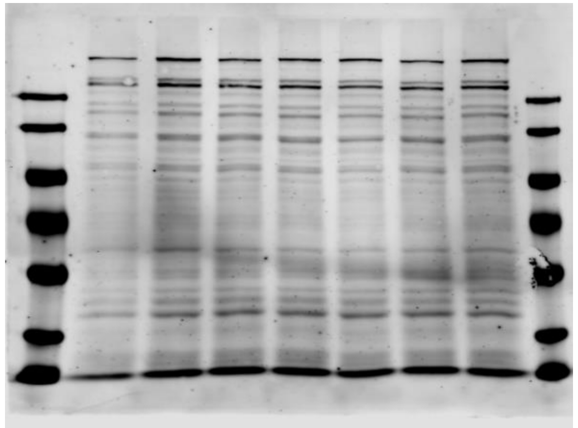

Repeat 2

OXY UT EGF 50 100 200

p-mTOR 289 kDa Rabbit

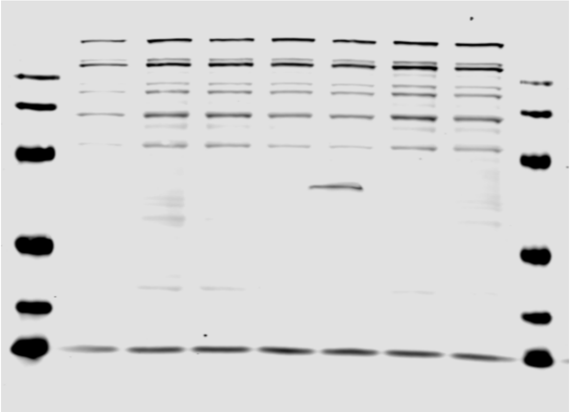

mTOR 289 kDa Rabbit

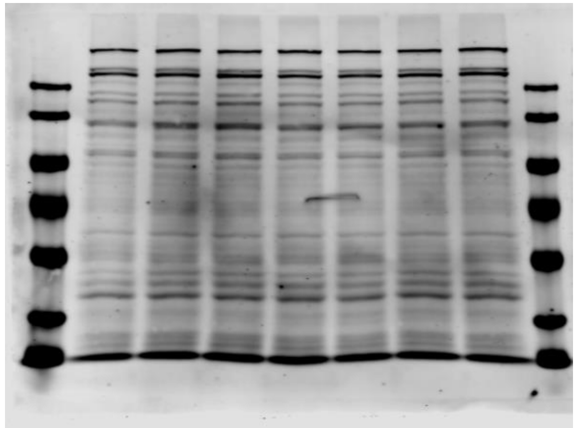

Repeat 3

OXY UT EGF 50 100 200

p-mTOR 289 kDa Rabbit

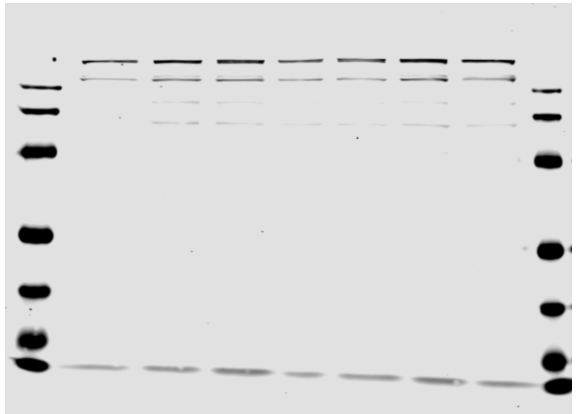

mTOR 289 kDa Rabbit

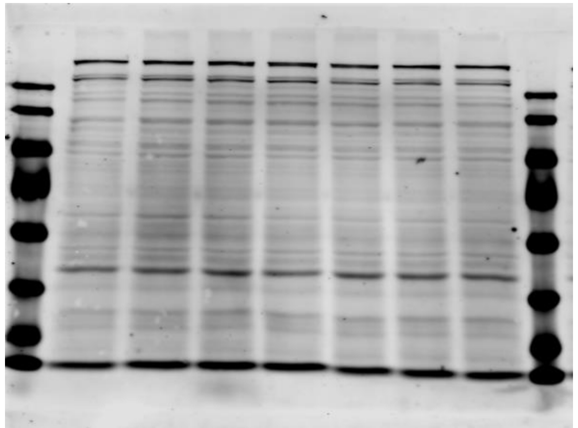

TOV21G

Cell apoptosis experiment

3 repeated results represented Fig 6

Repeat 1 | Repeat 2 | Repeat 3

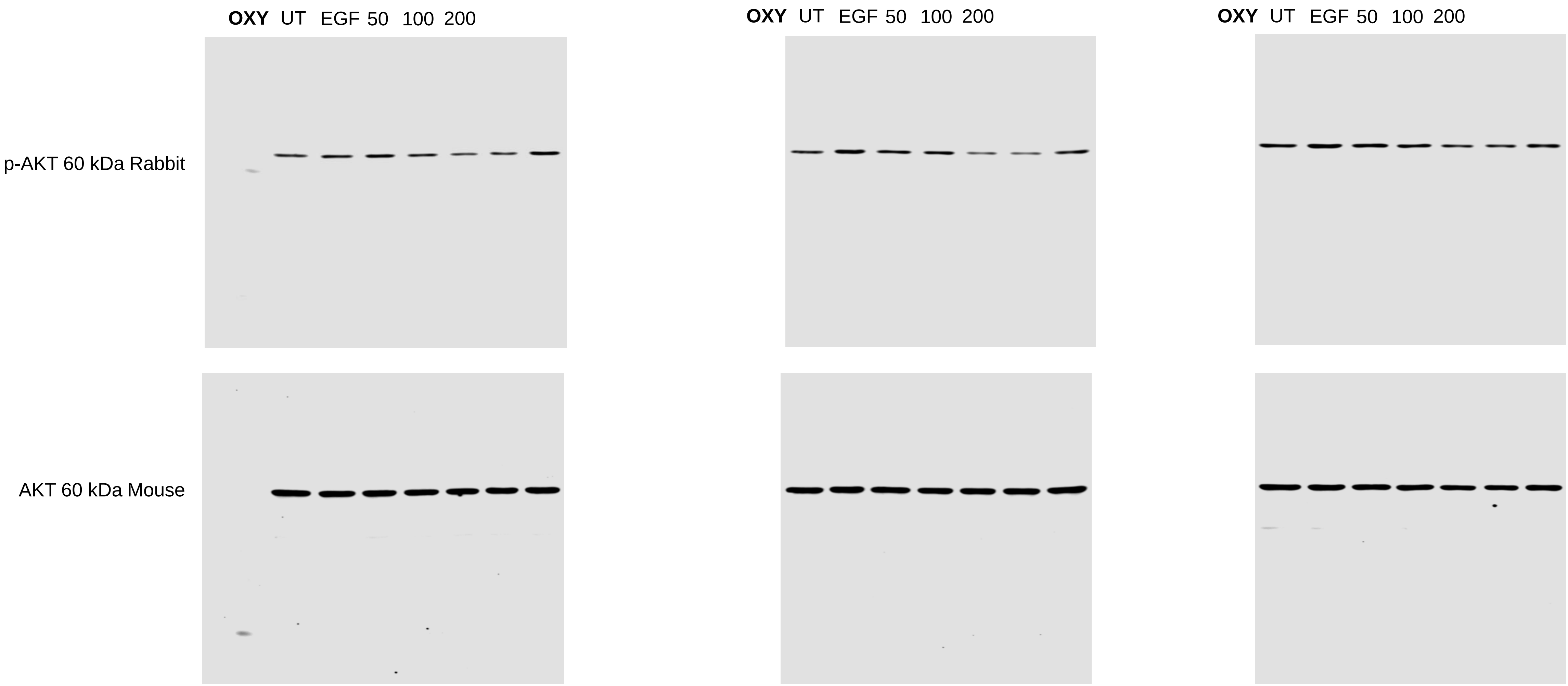

TOV21G

Cell apoptosis experiment

3 repeated results represented Fig 6

	Repeat 1	Repeat 2	Repeat 3
	OXY UT EGF 50 100 200	OXY UT EGF 50 100 200	OXY UT EGF 50 100 200

p-PDK1 175 kDa Rabbit

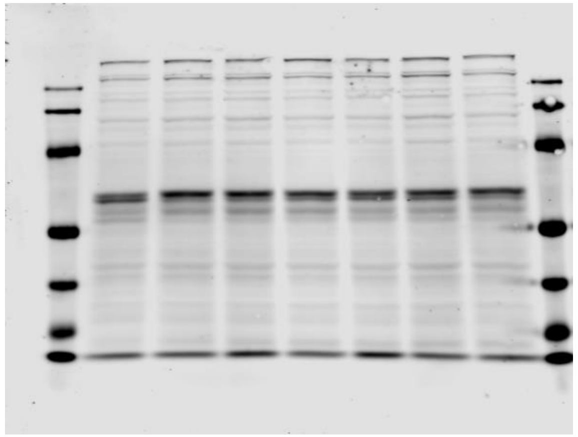

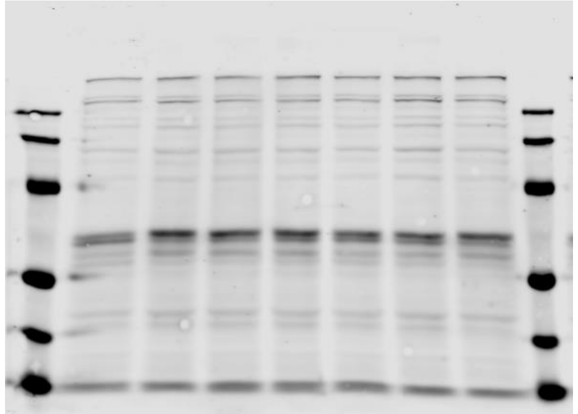

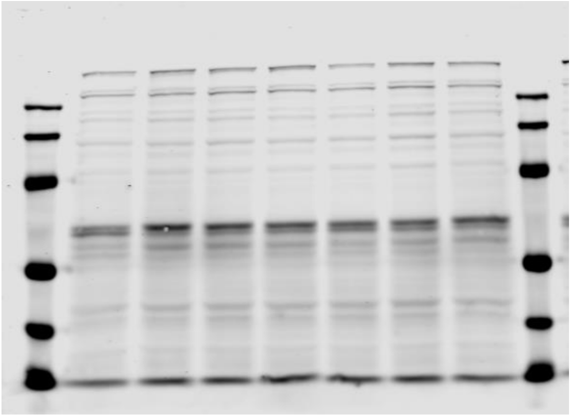

PDK1 175 kDa Rabbit

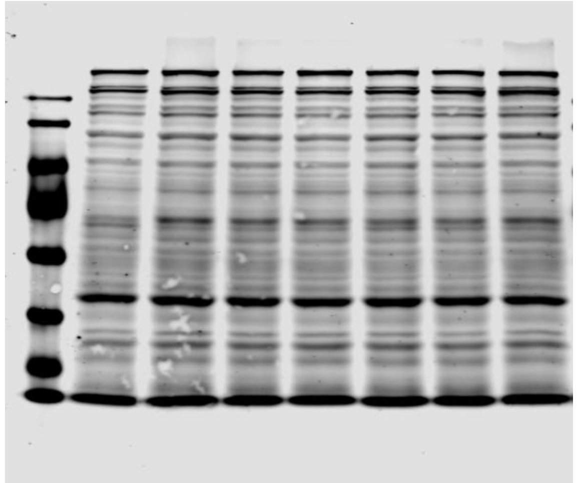

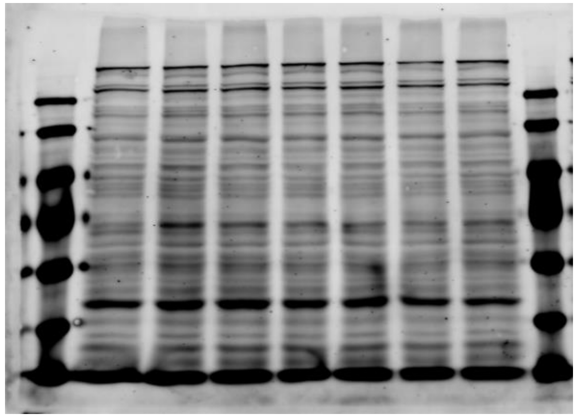

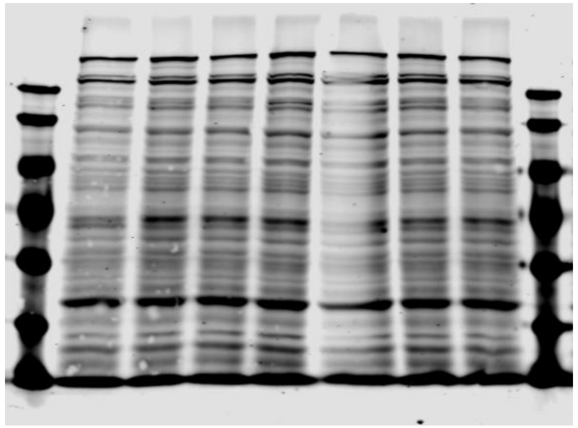

TOV21G

Cell apoptosis experiment

3 repeated results represented Fig 6

Repeat 1 | Repeat 2 | Repeat 3

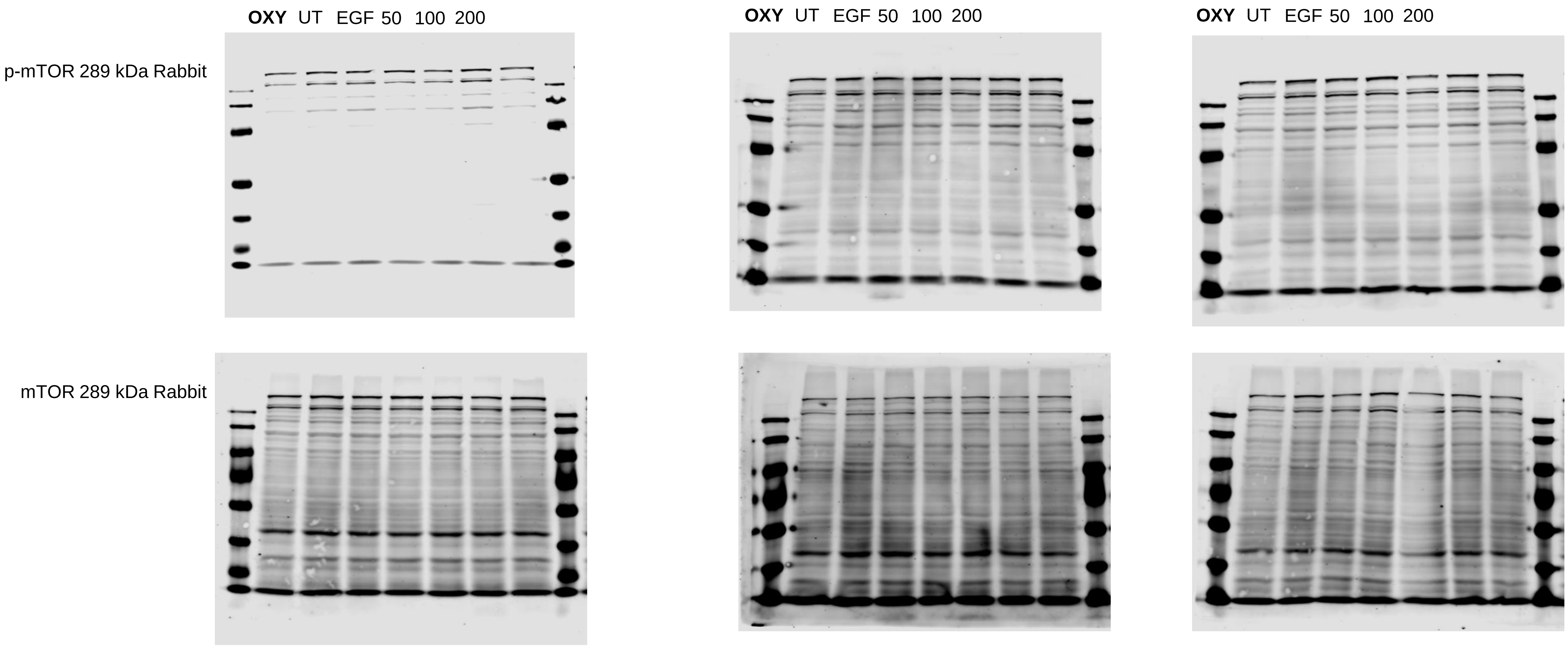

SKOV3

Cell apoptosis experiment

3 repeated results represented Fig 7

Repeat 1

OXY UT EGF 50 100 200

p-ERK1/2 42, 44 kDa Rabbit

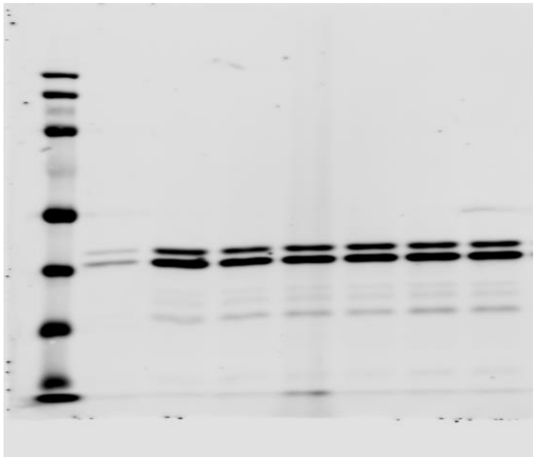

ERK1/2 42, 44 kDa Mouse

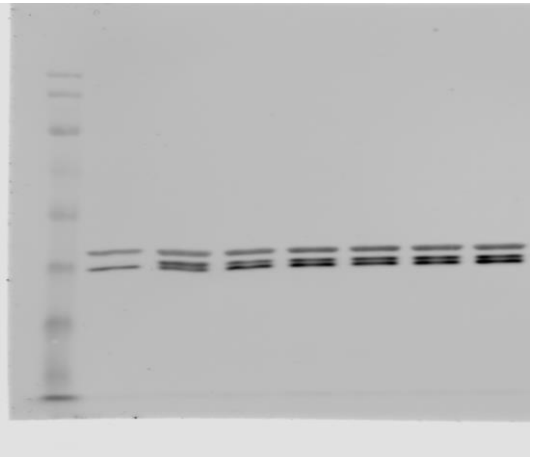

Repeat 2

OXY UT EGF 50 100 200

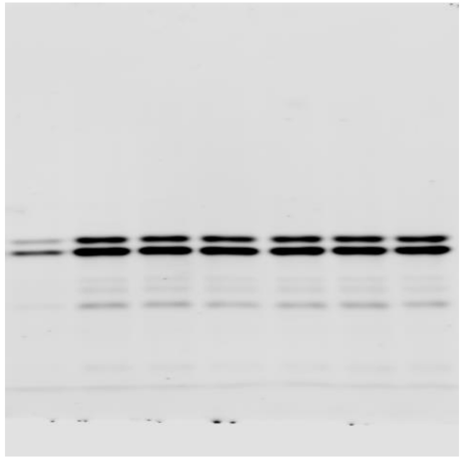

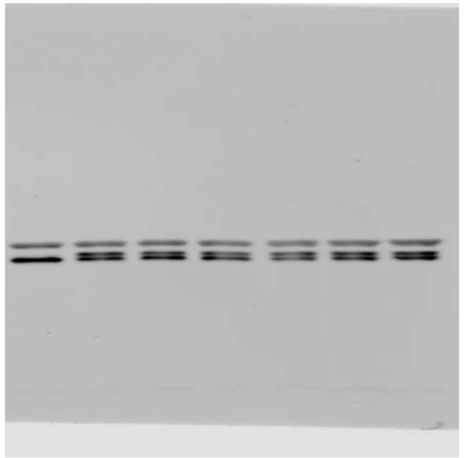

Repeat 3

OXY UT EGF 50 100 200

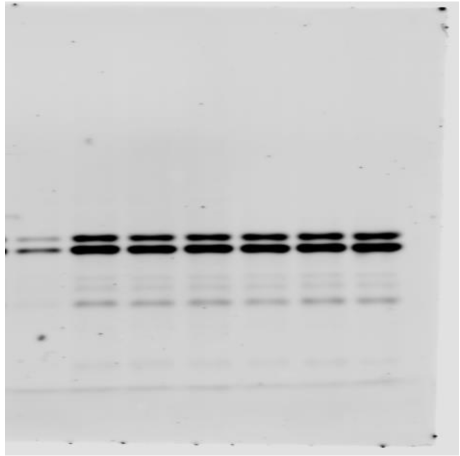

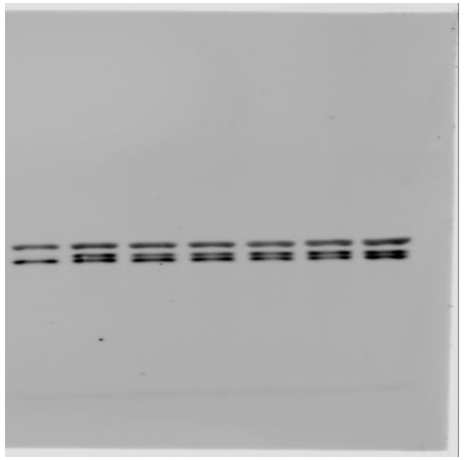

TOV21G

Cell apoptosis experiment

3 repeated results represented Fig 7

Repeat 1 **Repeat 2** **Repeat 3**

OXY UT EGF 50 100 200 | **OXY** UT EGF 50 100 200 | **OXY** UT EGF 50 100 200

p-ERK1/2 42, 44 kDa Rabbit

ERK1/2 42, 44 kDa Mouse

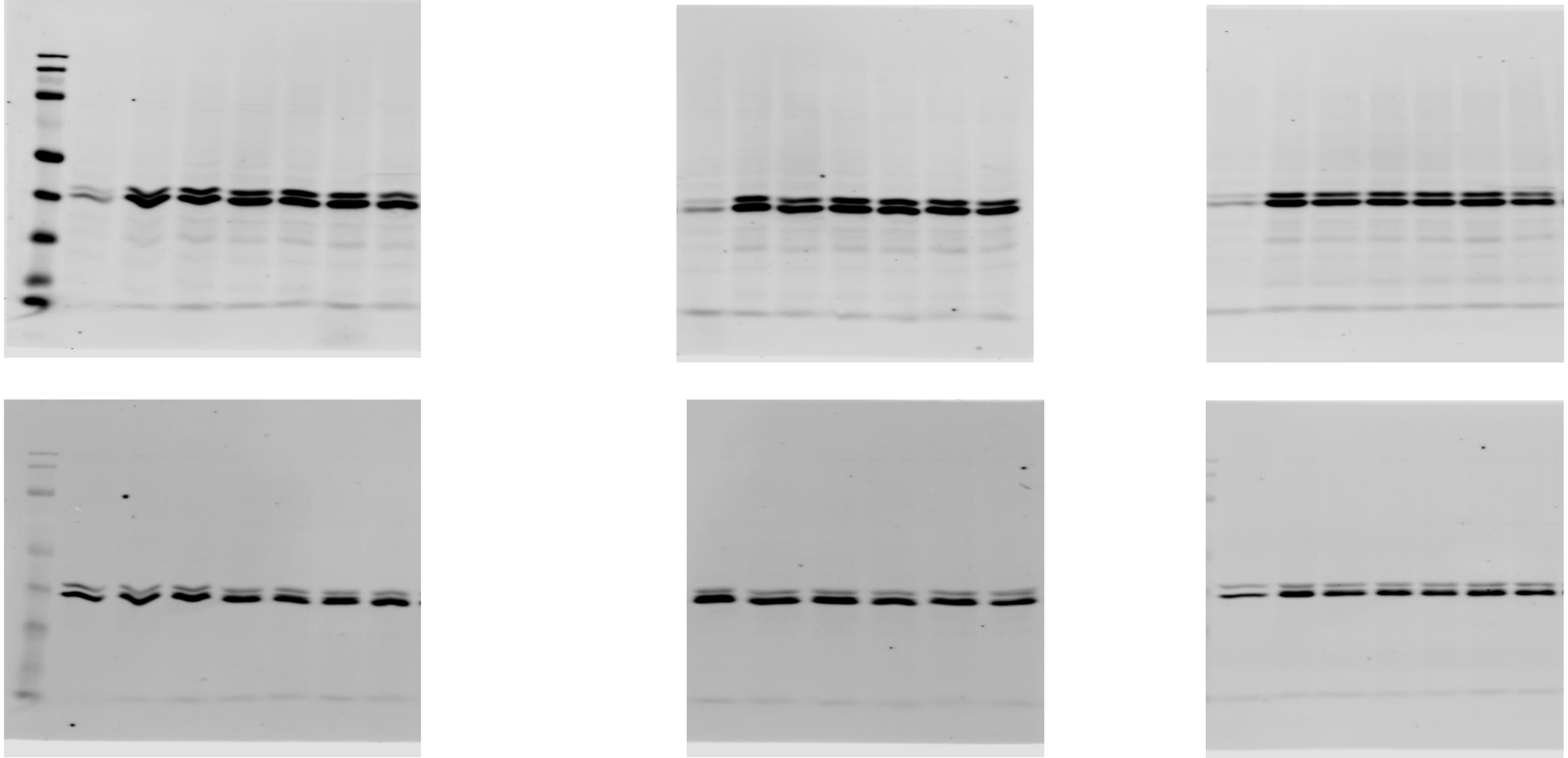

Supplement: Supplementary file 1 [file biomolecules-14-01140-s001.zip › biomolecules-3181272-original-images.pdf]
