# Supplementary material for: Oxyresveratrol Enhances the Anti-Cancer Effect of Cisplatin against Epithelial Ovarian Cancer Cells through Suppressing the Activation of Protein Kinase B (AKT)
Source: Biomolecules. 2024 Sep 9;14(9):1140. doi: 10.3390/biom14091140 (PMC11430010; doi:10.3390/biom14091140)
Supplement: Supplementary file 1 [file biomolecules-14-01140-s001.zip › biomolecules-3181272-supplementary.pdf]

Supplementary data

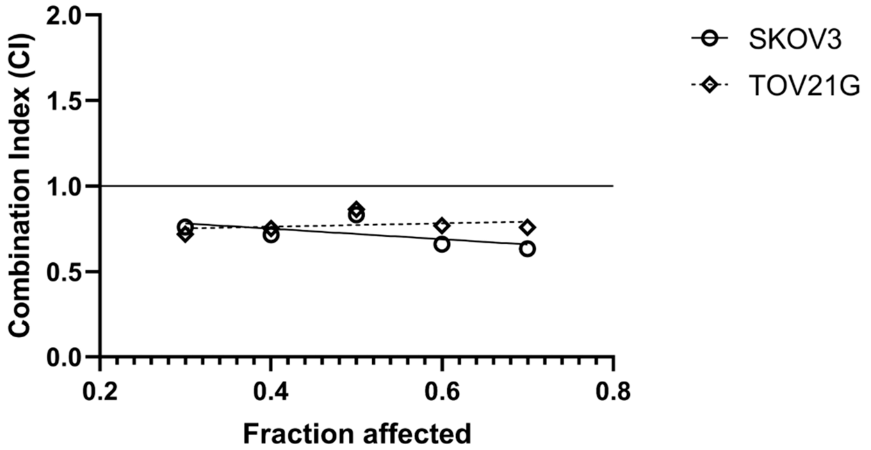

Combination index	Fraction affected				
	0.3	0.4	0.5	0.6	0.7
**SKOV3**	0.760	0.715	0.833	0.660	0.634
**TOV21G**	0.719	0.752	0.864	0.769	0.760

Supplement figure S1. The combination index of OXY and cisplatin co-treatment in cell viability at each fraction affected concentration in SKOV3 and TOV21G cells.
